# Supplementary material for: Sialidases and fucosidases of Akkermansia muciniphila are crucial for growth on mucin and nutrient sharing with mucus-associated gut bacteria
Source: Nat Commun. 2023 Apr 1;14:1833. doi: 10.1038/s41467-023-37533-6 (PMC10067855; doi:10.1038/s41467-023-37533-6)
Supplement: Supplementary file 1 — Supplementary Information [file 41467_2023_37533_MOESM1_ESM.pdf]

Supplementary Figures:

Supplementary Fig. 1: Modular organisation and sequence comparison of *A. muciniphila* fucosidases
Supplementary Fig. 2: Modular organisation of characterised GH29 and GH95 fucosidases
Supplementary Fig. 3: Phylogenetic clustering of *A. muciniphila* GH29 and GH95 fucosidases
Supplementary Fig. 4: Activity of *A. muciniphila* fucosidases on model oligosaccharides
Supplementary Fig. 5: Activity profiles of *A. muciniphila* fucosidases on selected mucin *O*-glycans and *N*-glycans
Supplementary Fig. 6: Fucosidase activity on defined *O*-glycoprotein conjugated Lewis epitopes
Supplementary Fig. 7: Modular organization and phylogeny of *A. muciniphila* sialidases
Supplementary Fig. 8: Sialidase activity on oligosaccharides
Supplementary Fig. 9: Sialidase activity on sialylated mucin *O*-glycan motifs and immunoglobulin G *N*-glycans
Supplementary Fig. 10: Cobra strike pose architecture and putative binding domains of *Am*GH29D
Supplementary Fig. 11: Comparison of the AlphaFold model of *Am*GH29C and the crystal structure of *Am*GH29D
Supplementary Fig. 12: Phylogenetic tree of GH181 as well as active site and surface binding site motif conservation
Supplementary Fig. 13: Architecture of *Am*GH181 and comparison to GH33 sialidases
Supplementary Fig. 14: Catalytic site signatures of *Am*GH181 as compared to the closest GH33 sialidase
Supplementary Fig. 15: The NMR analysis of the inverting mechanism of *Am*GH181
Supplementary Fig. 16: Ligand binding at the active site and secondary surface binding sites of *Am*GH181
Supplementary Fig. 17: Binding of *A. muciniphila* fucosidases and sialidases to mucin
Supplementary Fig. 18: Localisation of fucosidase and sialidase activities of *A. muciniphila*
Supplementary Fig. 19: Growth of butyrate-producing Clostridia on monosaccharides
Supplementary Fig. 20: Cross-feeding of butyrate-producing Clostridia in *A. muciniphila* co-cultures on mucins with different degree of sialyation

Supplementary Tables:

Supplementary Table 1: Enzyme names and primers
Supplementary Table 2: Kinetics parameters of *A. muciniphila* fucosidases
Supplementary Table 3: Number of assigned *O*- and *N*-glycan structures studied in this work
Supplementary Table 4: Fucosidase relative activity towards porcine gastric, colonic mucin and fetuin
Supplementary Table 5: Normalized activity of *A. muciniphila* fucosidases on HMOs, mucins and fetuin
Supplementary Table 6: Activity profiles of *A. muciniphila* sialidases on porcine colonic mucin *O*-glycans
Supplementary Table 7: Normalised activities of *A. muciniphila* sialidases on HMOs and attached *O*-glycans from mucin and fetuin
Supplementary Table 8: The top structural orthologues of *Am*GH29D
Supplementary Table 9: The top structural orthologues of *Am*GH181
Supplementary Table 10: The top structural orthologues of the CBM-like domain of *Am*GH181
Supplementary Table 11: Inhibition of *A. muciniphila* fucosidases by 1-Deoxyfuconojirimycin (DFJ)
Supplementary Table 12: Inhibition of *A. muciniphila* sialidases by 2,3-dehydro-2-deoxy-*N*-acetylneuraminic acid (DANA)
Supplementary Table 13: The effect of fucosidase and sialidase inhibition on growth of *A. muciniphila* on PCM
Supplementary Table 14: Prevalence of fucosidase and sialidase genes in *A. muciniphila* genomes
Supplementary Table 15: Data collection and refinement statistics for *Am*GH29D
Supplementary Table 16: Data collection and refinement statistics for *Am*GH181

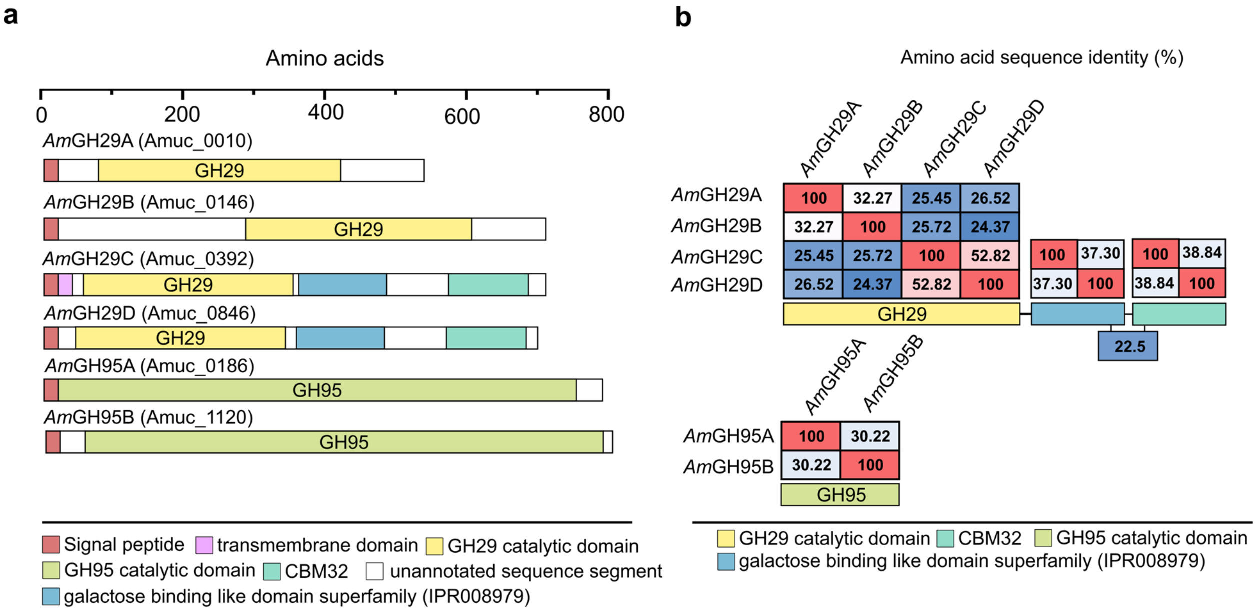

Supplementary Fig. 1: Modular organisation and sequence comparison of *A. muciniphila* GH29 and GH95. a, Size and modular organisation based on annotations by CAZy, dbCAN meta server, InterPro, and signal peptide predictions using SignalP (v.5.0). **b,** Amino acid sequence identity matrix showing the evolutionary relationships amongst the catalytic modules of *A. muciniphila* GH95 and GH29 enzymes and well as within the putative CBMs of the *A. muciniphila* GH29s.

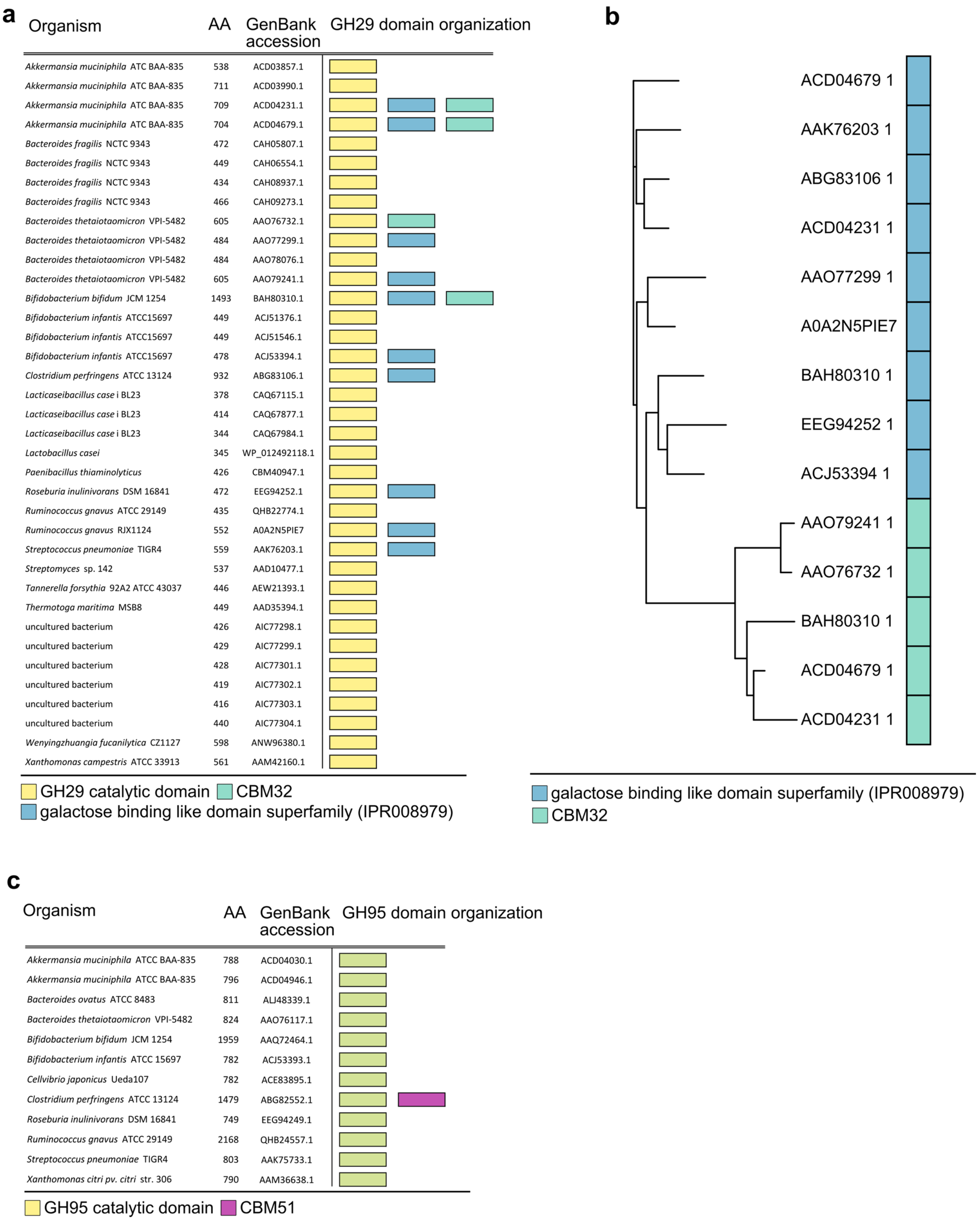

Supplementary Fig. 2: Modular organisation of characterized GH29 and GH95 fucosidases. a, Size and domain organisation of the four *A. muciniphila* GH29s and of previously characterized GH29 enzymes. **b,** Phylogenetic analysis showing the segregation of the galactose-binding-like domains and the CBM32 that occur in previously characterized GH29s and in *A. muciniphila* GH29 enzymes described in the present study. **c,** Size and modular organization of the two *A. muciniphila* GH95 fucosidases and of hitherto characterized GH95 enzymes. Domain annotations are from CAZy, dbCAN meta server and InterPro.

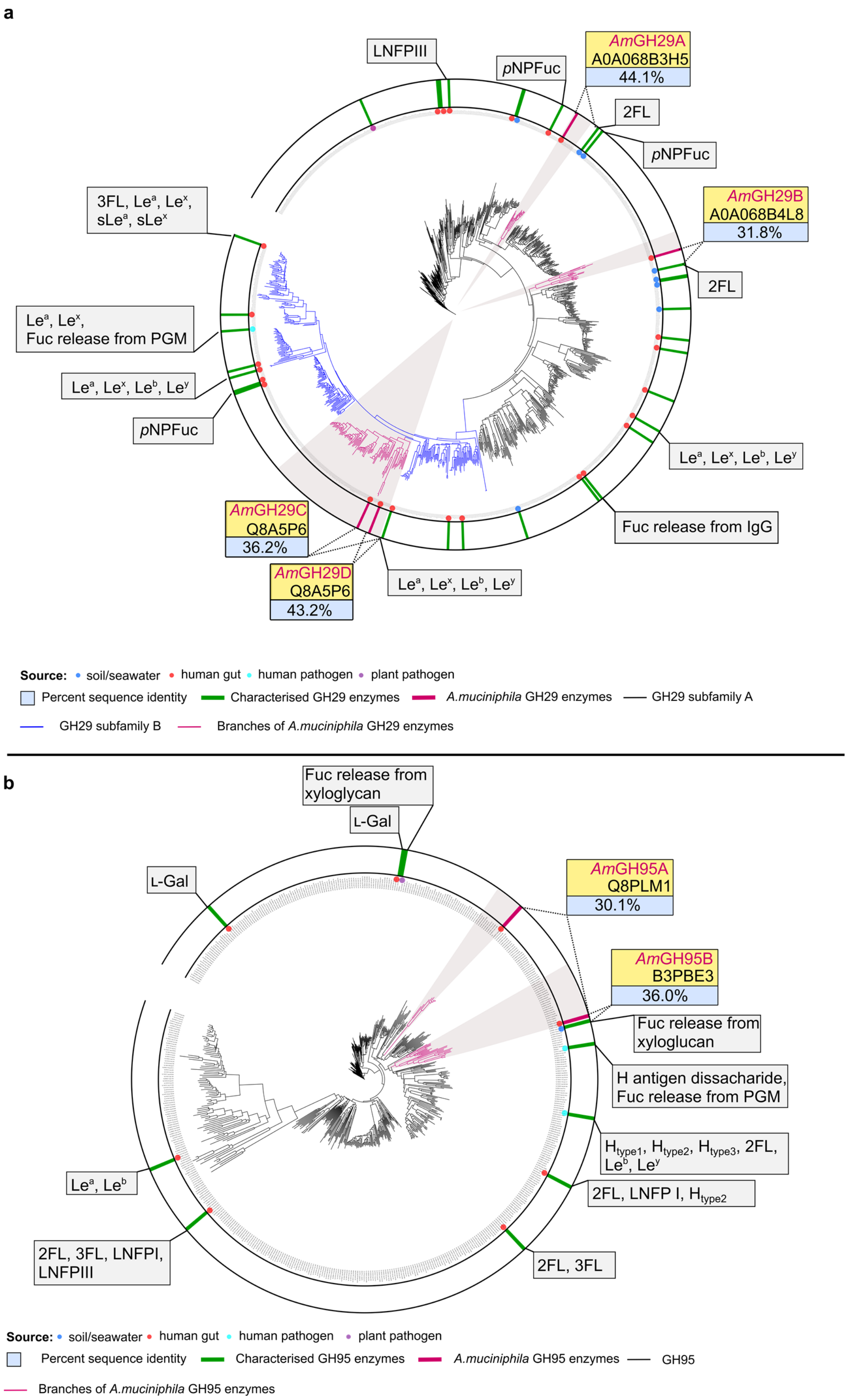

Supplementary Fig. 3: Phylogenetic clustering of the *A. muciniphila* GH29 and GH95 fucosidases. a, Phylogenetic tree of 1117 putative GH29 sequences. **b,** Phylogenetic tree of 543 putative GH95 sequences. Characterized GH29s and GH95s (as defined in CAZy) are green stripes, *A. muciniphila* GH29 and GH95 enzymes are in magenta stripes. *A. muciniphila* fucosidases and their closest characterized orthologues (indicated with their UniProt IDs) are in yellow boxes and the amino acid sequence identities between their catalytic modules are in the blue box. The source niches of the described fucosidases are indicated by coloured circles and the substrates the enzymes have been shown to be active on are shown. The sequences belonging to GH29 subfamily A (high activity on *p*NPFuc) and B (low activity on *p*NPFuc) are in black and blue, respectively. The branches populated by the *A. muciniphila* enzymes are in pink and highlighted by a grey shadow.

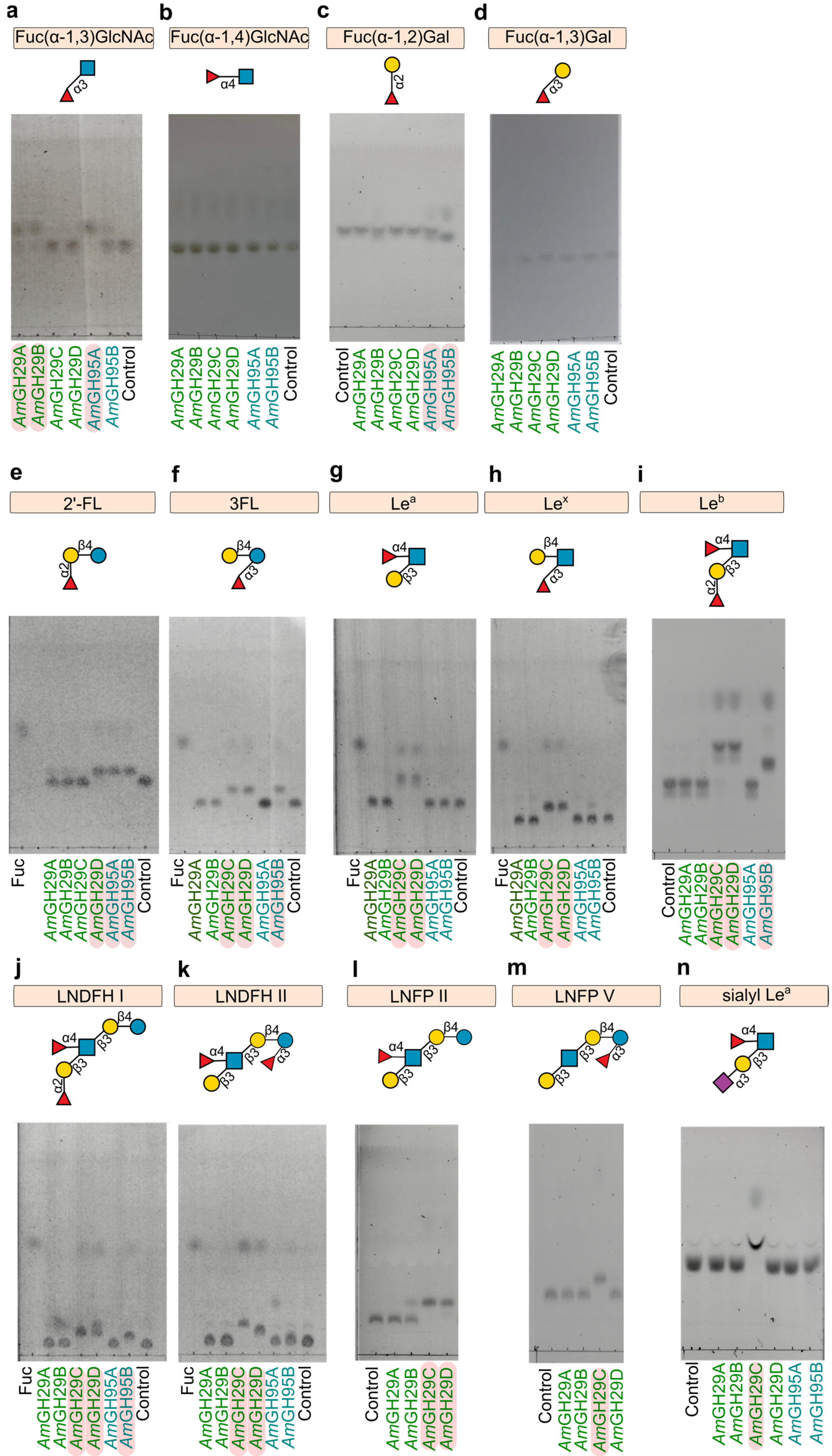

Supplementary Fig. 4: The activity of *A. muciniphila* fucosidases on model oligosaccharides. a-n, Fucosidase activity analysed using thin layer chromatography on di- and oligosaccharides using 2 mM substrate, 0.5 µM enzyme, pH 6.8 at 37 °C for 1 h. The enzymes that display activity are highlighted by a pink box. The data were from 3 independent experiments (n=3), whereby the triplicate analyses yielded similar results. Source data are provided as a Source Data file labelled with the corresponding figure number and panel definition.

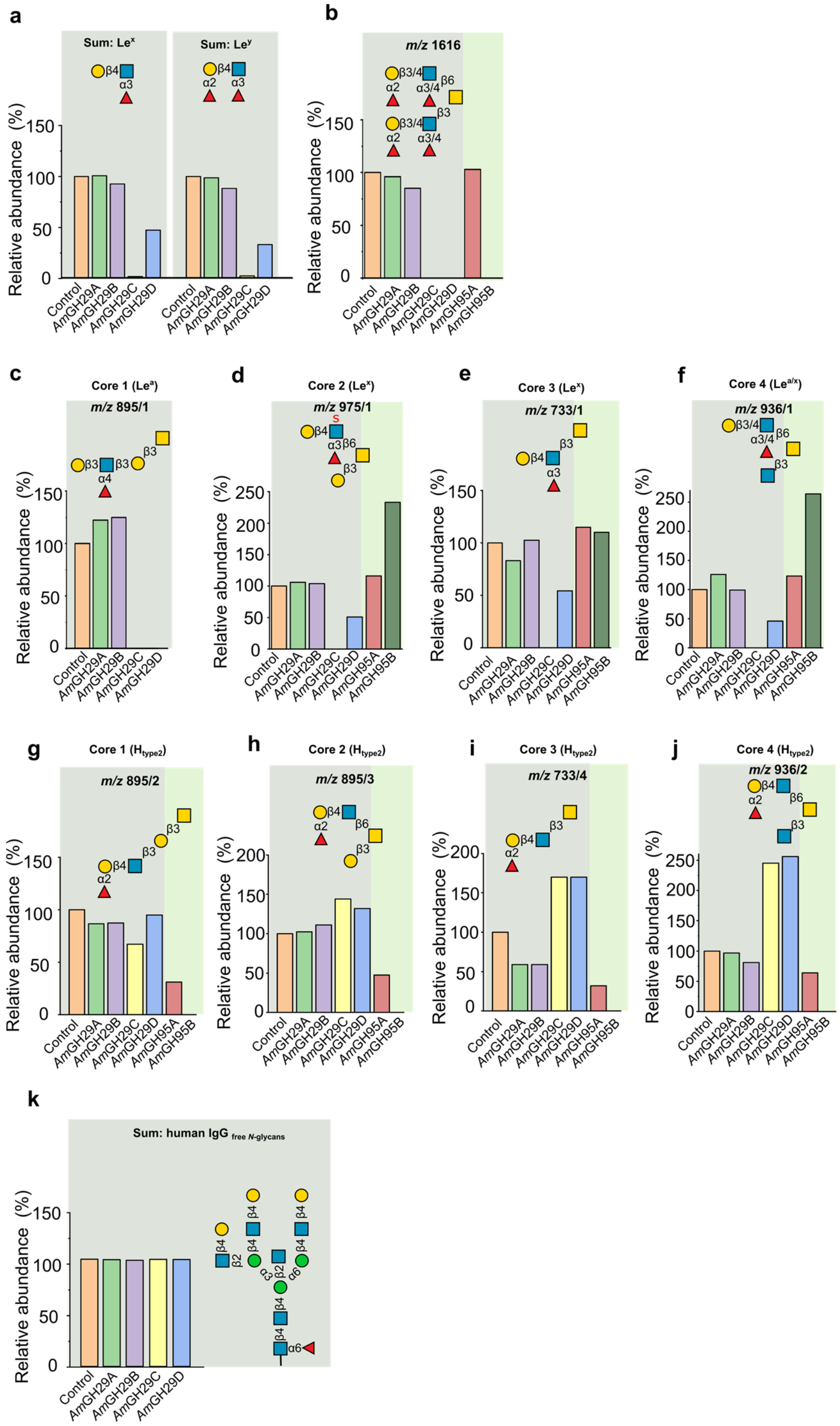

Supplementary Fig. 5: Activity profiles of *A. muciniphila* fucosidases on selected mucin *O*-glycans and *N*-glycans. The *O*-glycans were conjugated to colonic mucin, whereas the *N*-glycans were enzymatically released prior to incubation with enzymes. Both GH95 enzymes are α1,2-specific and only motifs containing such linkages are shown. **a,** Activity profiles showing the tolerance of adjacent double fucosylation. **b,** Activity profiles showing the tolerance of double branch fucosylations. **c,** Activity profiles showing the tolerance on the Le^{a} epitope on core 1 structures. **d,** Activity profiles of *Am*GH29C and *Am*GH29D on core 2 structures. **e,** Activity profiles of *Am*GH29C and *Am*GH29D on core 3 structures. **f,** Activity profiles of *Am*GH29C and *Am*GH29D on core 4 structures. **g,** Activity profiles of *Am*GH95A and *Am*GH95B on core 1 structures. **h,** Activity profiles of *Am*GH95A and *Am*GH95B on core 2 structures. **i,** Activity profiles of *Am*GH95A and *Am*GH95B on core 3 structures. **j,** Activity profiles of *Am*GH95A and *Am*GH95B on core 4 structures. **k,** Lack of activity towards α1,6-fucosyl, present in human *N*-glycans. For isobaric glycans structures, the number after the slash (/n) denotes the corresponding structure in the LC-MS data file (see Supplementary Data File 1). Following enzyme incubations, the *O*-glycans were released by reductive amination and analysed by ESI-LC MS/MS. Data are from a single ESI-LC MS/MS (n=1) analysis. For comparing the relative abundance of glycans between samples, the AUC (ESI- LC MS/MS ion chromatogram peak area) of each glycan structure was integrated, normalized to the total AUC of the sample and expressed as a percentage. The increase in relative abundance of structures presented in **d** and **f** originates from the activity of *Am*GH95B on the Le$^{b/y}$ precursor glycans with a $m/z_{precurser}$=1121-1 (for **e**) and $m/z_{precurser}$=1082 (for **f**) whereas the increase in relative abundance of structures presented in **i** and **j** results from the activity of *Am*GH29C and *Am*GH29D on the Le^{y} precursor glycans with a $m/z_{precurser}$=879 (for **i**) and $m/z_{precurser}$=1082 (for **j**). A Source Data file labelled with the corresponding figure number and panel definition is available.

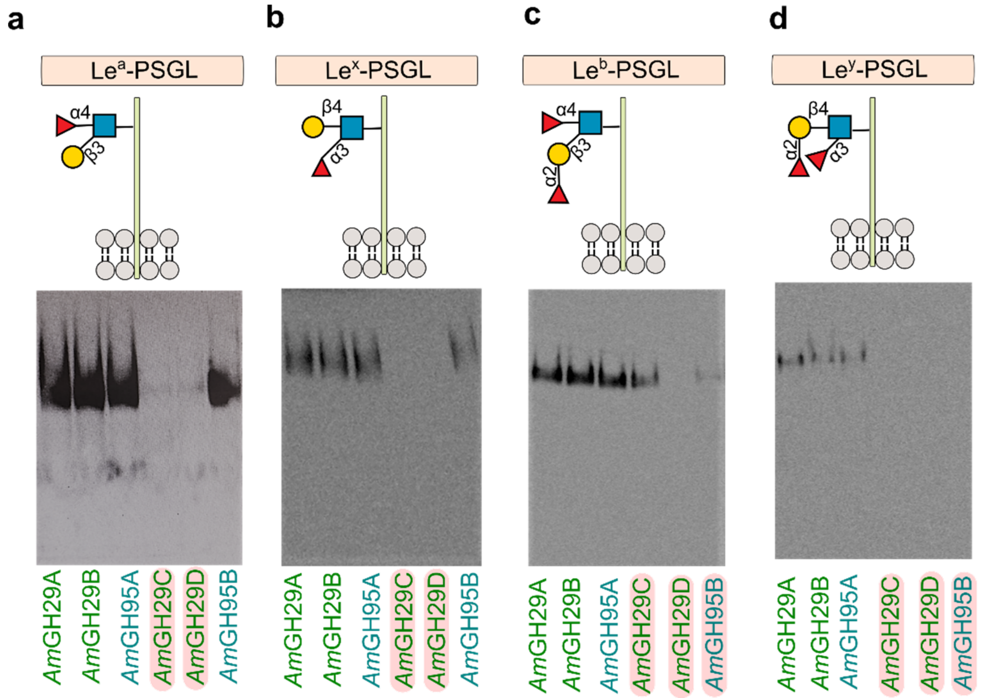

Supplementary Fig. 6: Fucosidase activity on defined *O*-glycoprotein conjugated Lewis epitopes. a-d, Fucosidase activity is observed as a decrease/loss of Western blot signal on defined conjugated Lewis epitopes presented by the recombinant glyco-engineered P-selectin glycoprotein ligand-1 (PSGL1) from engineered CHO cells. Each enzyme (2 µM) was incubated with beads carrying PSGL-1 glycoprotein (displaying a specific Le antigens) in 20 mM HEPES buffer 150 mM NaCl pH 6.8 at 37 °C for 3 h in 50 µl. The beads were boiled in presence of SDS-loading buffer containing 25 mM DTT for 10 min at 95°C and western blot analysis was performed (see materials and methods). For simplicity, only the defined Lewis epitopes of the native protein *O*-glycome are shown. The enzymatic activity is monitored by the loss of Western blot signal originating from specific Le-epitope antibodies after enzyme incubation. The cleavage of a fucosyl would be observed as a loss/decrease of signal due to the large decrease/abolished binding of the antibody. Inactive enzymes serve also as negative controls as they show independently the full-signal, whereas the active enzymes serve as positive controls, showing signal level when the motif is depleted. The data are from a single (n=1) experiment. Source data are provided as a Source Data file labelled with the corresponding figure number and panel definition.

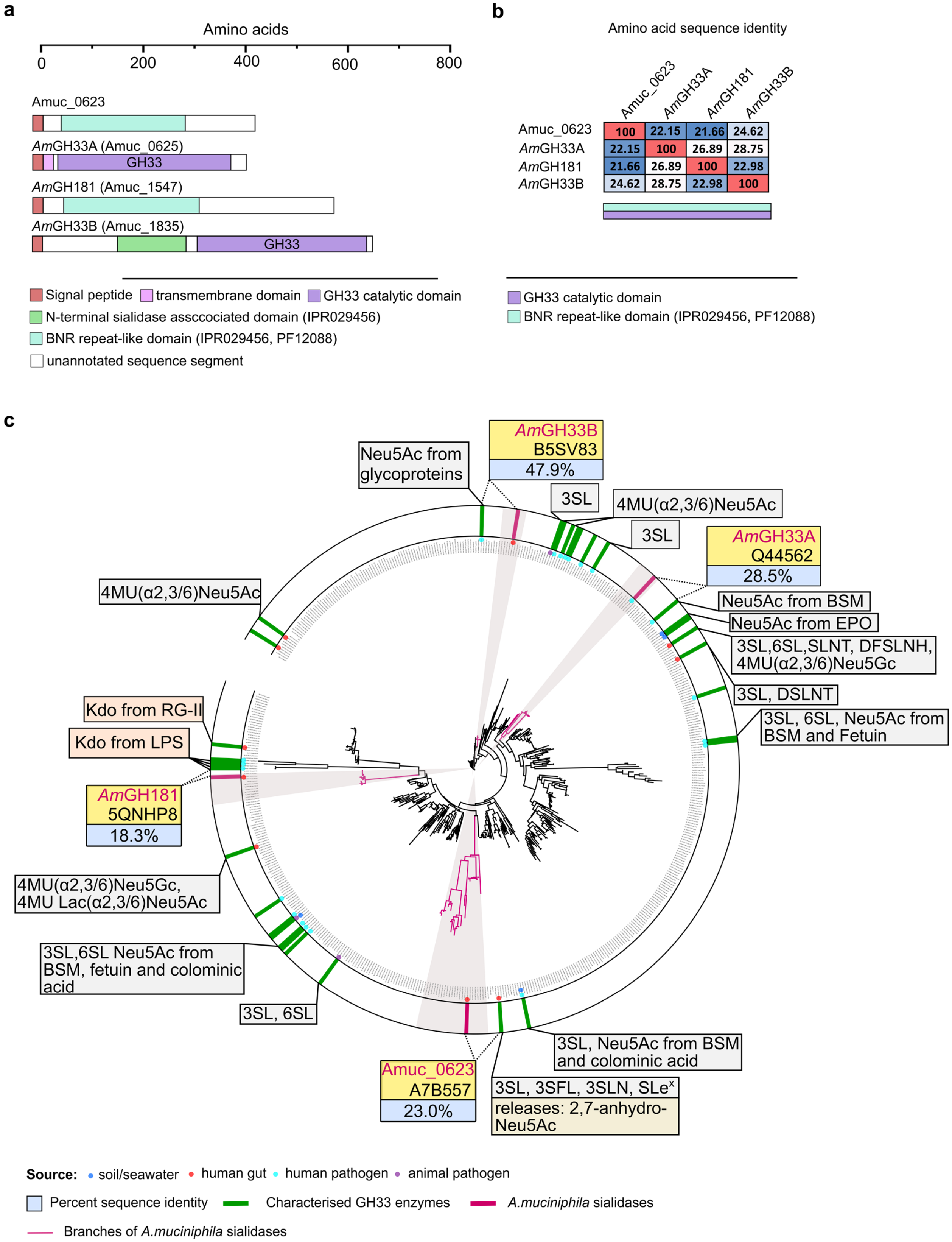

Supplementary Fig. 7: Modular organization and phylogeny of *A. muciniphila* sialidases. a, Modular organization of *A. muciniphila* assigned GH33 sialidases and distant orthologues with BNR-like domains, considered a sialidase signature. **b,** Amino acid sequence identity matrix for the sequences in a**. c,** Phylogenetic tree of 534 putative sialidases sequences (GH33 enzymes and BNR-like domain enzymes from the sequences in a and b). *A. muciniphila* sialidases and their closest characterized orthologues (indicated with their UniProt IDs) are in yellow boxes and the amino acid sequence identities between their catalytic modules are in the blue box. The source organism niches of the described sialidases are indicated by coloured circles and substrates that enzymes have been shown to be active on are shown. The branches populated by the *A. muciniphila* enzymes are in pink and highlighted by a grey shadow. Characterized GH33 enzymes previously shown to release 2,7-anhydro Neu5Ac are highlighted by a dark yellow box and GH33 enzymes demonstrated to be active on 2-keto-3-deoxy-D-manno-octulosonic acid (Kdo) are highlighted by an orange box.

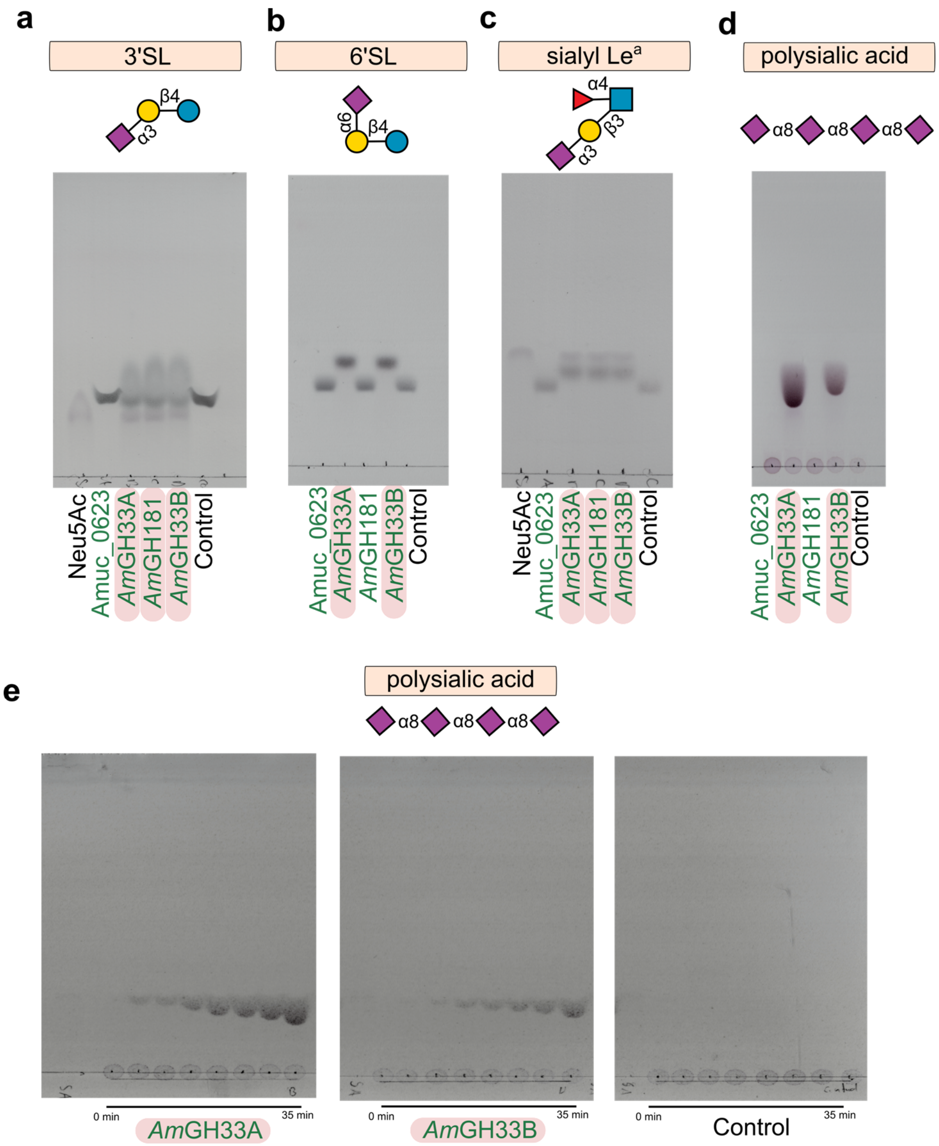

Supplementary Fig. 8: Sialidase activity on oligosaccharides. a-e, The sialidase activity on sialyl-substituted oligosaccharides and polysialic acid (Colominic acid) monitored by TLC. Active enzymes are highlighted by pink boxes. Reactions (10 µL) were carried out using 2 mM of each substrate, 0.5 µM of each enzyme at pH 6.8 at 37 °C for 1 h. The data are from three independent experiments (n=3), whereby all analyses yielded similar results. Source data are provided as a Source Data file labelled with the corresponding figure number and panel definition.

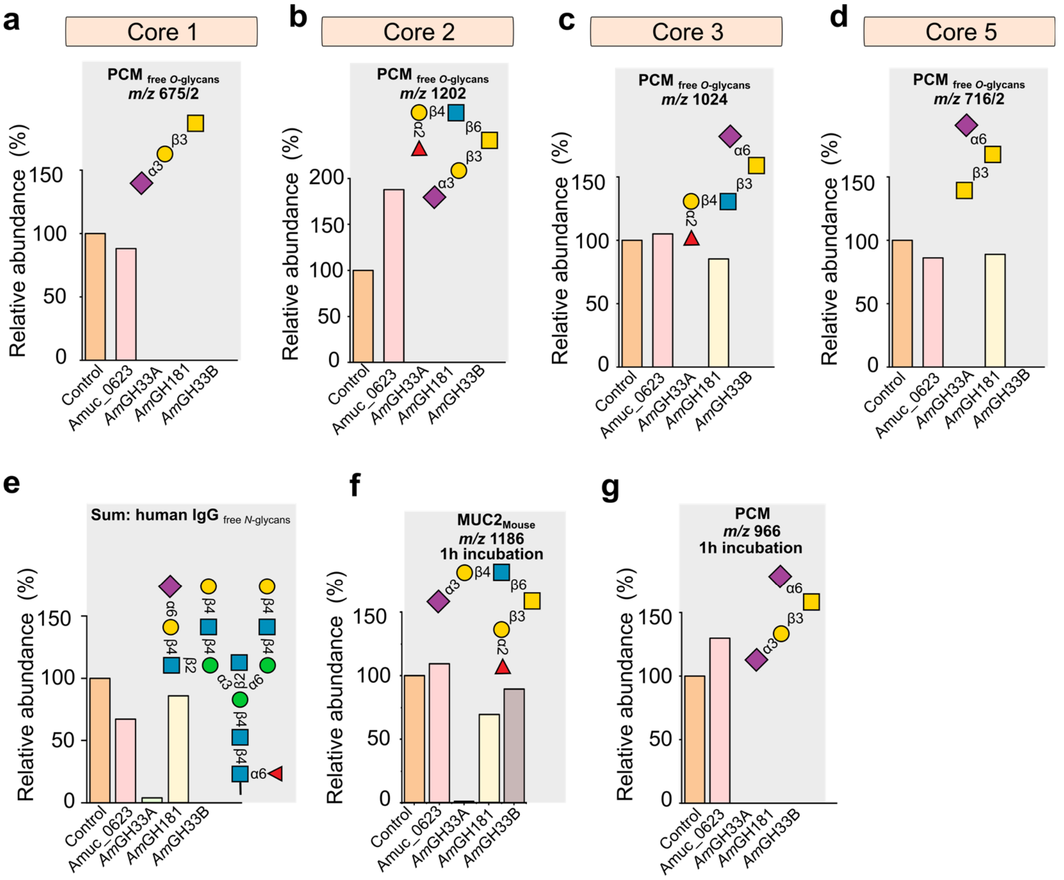

Supplementary Fig. 9: Sialidase activity on mucin sialylated *O*-glycan motifs as well as immunoglobulin G *N*-glycans. The subscripted "free *O*-glycans" indicated that the analyses were performed on released glycans, whereas absence of this subscript refers to analyses conducted on *O*-glycans attached to mucin **a-d,** Activity on different *O*-glycan cores from porcine colonic mucin (PCM). **e,** Sialidase activity on *N*-glycans from human IgG. **f,** Activity profiles showing equal activity of *Am*GH33A on the 6SGalNac epitope from PCM and $Muc2_{Mouse}$ (Muc2 from mouse) after 1 h incubation, while the activity of *Am*GH33B on the same epitope is markedly lower on intact $Muc2_{Mouse}$. **g,** Activity profile showing the lower activity of *Am*GH33B on the Sd^a epitope from $Muc2_{Mouse}$ as compared to the activity of the enzyme on the same epitope from PCM after 1 h incubation. For isobaric glycans structures, the number after the slash (/n) denotes the corresponding structure in the LC-MS data file (see Supplementary Data). Either free *O*-glycans and *N*-glycans or MUC2 conjugated *O*-glycans were blotted on PVDF membranes and incubated with the enzymes for 1 h or 24 h (see material and methods for details). Following the incubations, the free glycans were analysed directly, whereas the conjugated glycans were released by reductive amination and analysed using ESI-LC MS/MS. The data are from a single (n=1) experiment. For comparing the relative abundance of glycans between samples, the AUC (ESI- LC MS/MS ion chromatogram peak area) of each glycan structure was integrated, normalized to the total AUC of the sample and expressed as a percentage. Relative abundances higher than the control are likely due to noise resulting from minor differences in mucin spotted amounts. For minor structures, this could result in large changes as some of the minor structures would not be detected in the control. This artefact does not change the overall activity profiles as verified by removing data from glycans with a relative abundance <0.5% (See Supplementary Data File 2). Source data are provided as a Source Data file labelled with the corresponding figure number and panel definition.

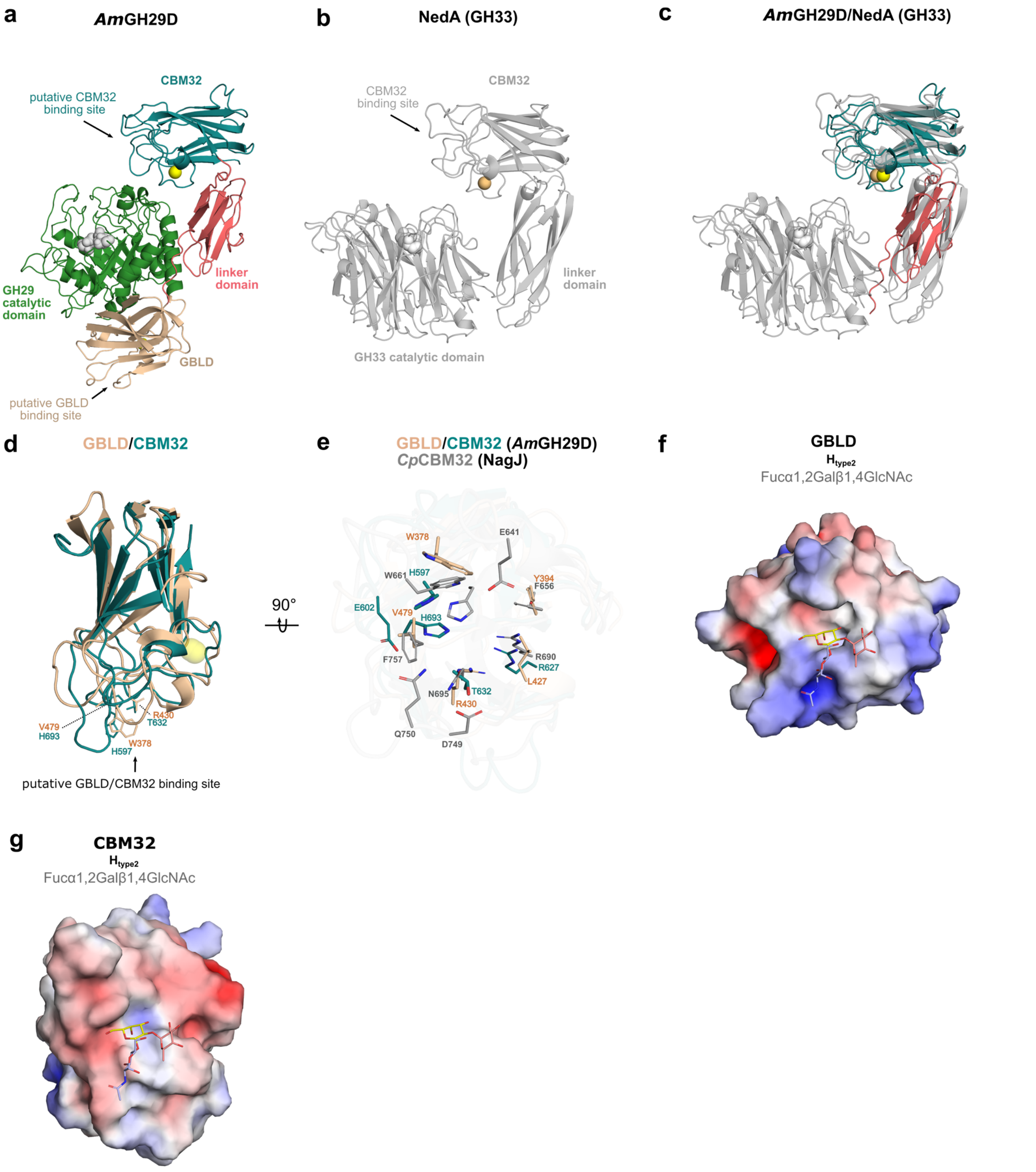

Supplementary Fig. 10: Cobra strike pose architecture and putative binding domains of *Am*GH29D. a, Overall structure of *Am*GH29D consisting of a catalytic $(\beta/\alpha)_8$ N-terminal domain (green), a β-sandwich forming the galactose binding like domain (GBLD) (wheat coloured), a linker domain (salmon red) and a putative CBM32 (dark cyan) with a Ca^{2+} (yellow sphere) binding site. The inferred catalytic nucleophile D190 and the acid/base E246 are shown as spheres (white). **b,** Overall structure of the *Micromonospora viridifaciens* GH33 sialidase (NedA, PDB: 1WCQ) that displays a similar "Cobra strike pose" architecture as *Am*GH29D. The catalytic residues are highlighted as spheres (grey) and the bound Na^{+} is shown as orange sphere, **c,** Structural alignment (Dali server) of NedA with the linker and CBM32 domain of *Am*GH29D showing a similar juxtapositioning above the active site (here dubbed as a Cobra strike pose). **d,** Structural alignment of the GBLD and the CBM32 domains of *Am*GH29D with the bound Ca^{2+} (yellow) and with residues putatively involved in ligand binding represented as sticks. **e,** Comparison of the ligand-binding sites of the biochemically characterized CBM32 from *Clostridium perfringens* (*Cp*CBM32, PDB: 2J7M) with GBLD and the CBM32 from *Am*GH29D. The analysis shows that the aromatic stacking tryptophan in the previously characterized CBM32 is shared with GLBD, which otherwise possesses more apolar residues in the potential binding site as compared to both CBM32s. **f,** Electrostatic surface representation of the GLBD (generated using the APBS plugin in Pymol) illustrating the apolar surface of the putative binding site. **g,** Electrostatic surface representation of the putative CBM32 showing a more polar putative binding site as compared to the GLBD (g). (**f** and **g)** The H2 antigen trisaccharide bound in *Cp*CBM32 with fucose shown in pink, *N*-acetylglucosamine in blue and galactose in yellow is also shown in the GBLD and CBM32 from *Am*GH29D after structural alignment with the *Cp*CBM32 (2J7M) to visualize the putative binding site area.

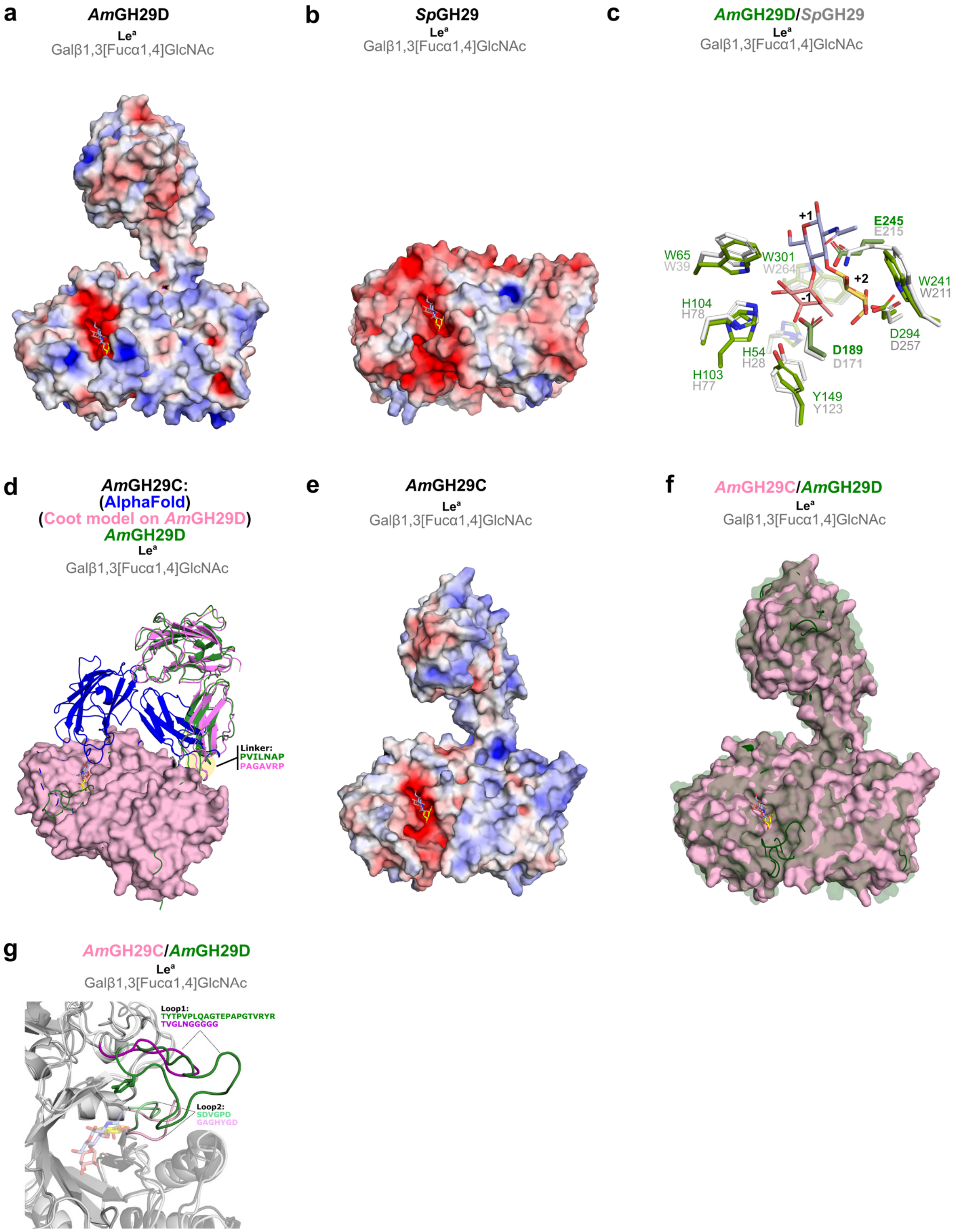

Supplementary Fig. 11: Comparison of the AlphaFold model of *Am*GH29C and the crystal structure of *Am*GH29D. a, Surface electrostatic potential of *Am*GH29D (APBS plugin in Pymol) showing the negatively charged active site surrounded by positively charged patches. **b,** Surface electrostatic potential of the closest structural orthologue of *Am*GH29D (*Sp*GH29, 6OR4;) showing a negatively charge patch flanking the active site as opposed to *Am*GH29D. **c,** Stick representation (green) of the *Am*GH29D active site shows a highly conserved −1 subsite as compared to *Sp*GH29 (white sticks). The catalytic residues of *Am*GH29D (D190 and E246) are in bold font. **d,** Superimposition of the *Am*GH29C AlphaFold model (pink surface (catalytic domain) and the C-terminal GBLD and CBM32 in cartoon), an *Am*GH29C model build in coot (blue, cartoon) on *Am*GH29D as a template (blue, cartoon) and *Am*GH29D (green, cartoon). Amino acid differences in the linker region suggest higher flexibility of *Am*GH29C than *Am*GH29D, which may contribute to the possible ridged body movement of the CBM32-linker domains in *Am*GH29C to position the CBM in a Cobra-bite pose at the side of the active site. **e,** Electrostatic surface representation of the AlphaFold model of *Am*GH29C. **f,** Superimposition of the *Am*GH29C Coot model (white solid surface) with *Am*GH29D (green semi-transparent surface) showing large loops in *Am*GH29D occluding the active site, potentially hindering the accommodation of larger complex and/or heavily substituted fuco-*O*-glycans. **g,** Superimposition of the *Am*GH29C AlphaFold model (dark grey, cartoon) with *Am*GH29D (white, cartoon) highlighting the larger and potentially less flexible loops in *Am*GH29D (Loop1: dark green, Loop2: light green) restricting the active site as opposed to the shorter and more flexible corresponding loops in *Am*GH29C (Loop1: dark pink, Loop2: light pink). The structure is shown from a different perspective for clarity.

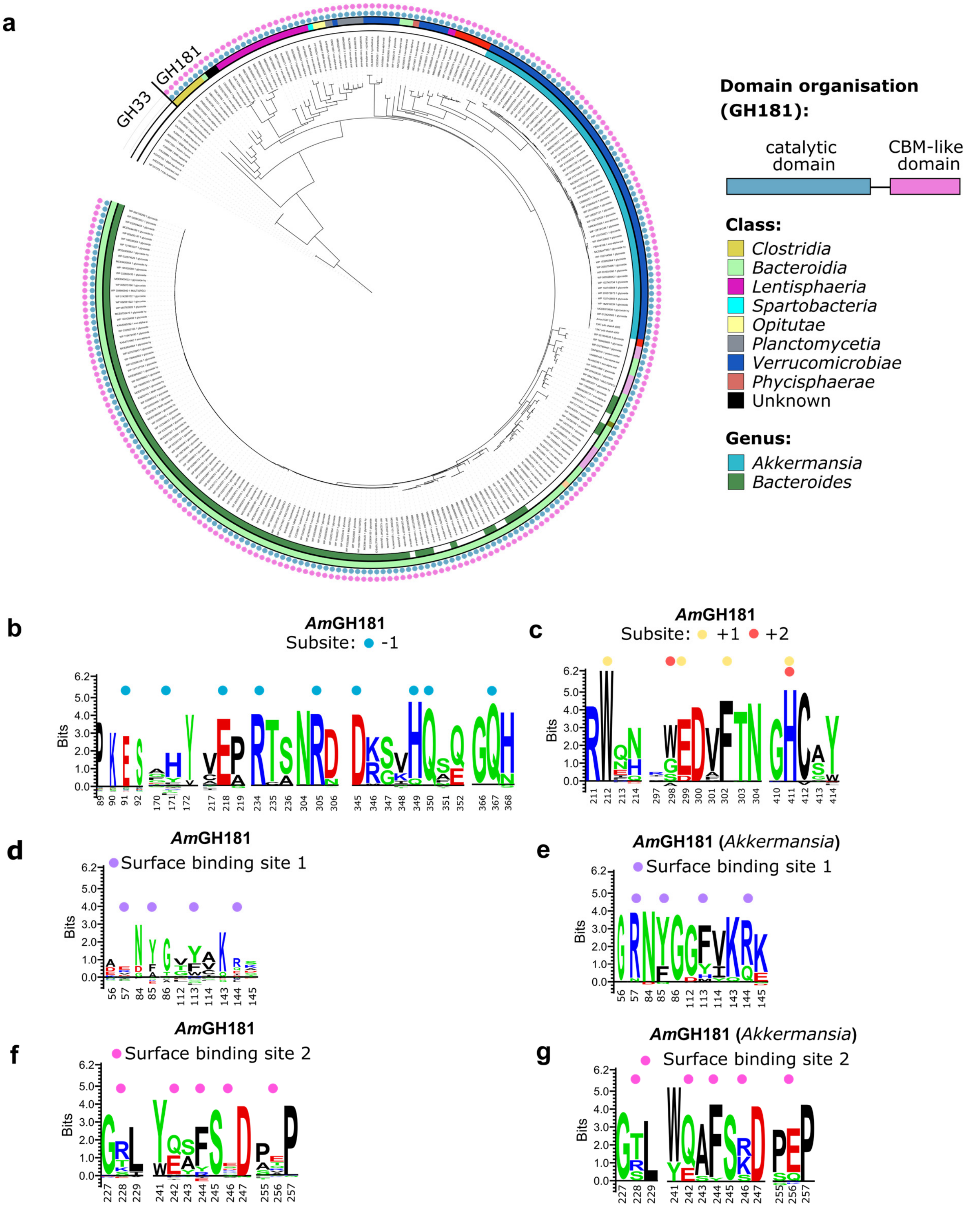

Supplementary Fig. 12: Phylogenetic tree of GH181 as well as active site and surface binding site motif conservation. a, Phylogenetic tree of 335 GH181 sequences generated using *Am*GH181 as a query (see Materials and methods section). The modular architecture of the catalytic module and the CBM-like domain is conserved throughout the family and the taxonomic class and genus affiliations of the sequences is shown. **b,** Sequence logo of subsites +1 and +2 subsites. **c,** Sequence logo of subsite -1. **d,** Sequence logo of the putative surface binding site 1 residues across GH181. **e,** same as **d**, but including only GH181 sequences from the *Akkermansia* genus. **f,** Sequence logo of the putative surface binding site 2 across GH181. **g,** same as **f**, but including sequences only from the *Akkermansia* genus.

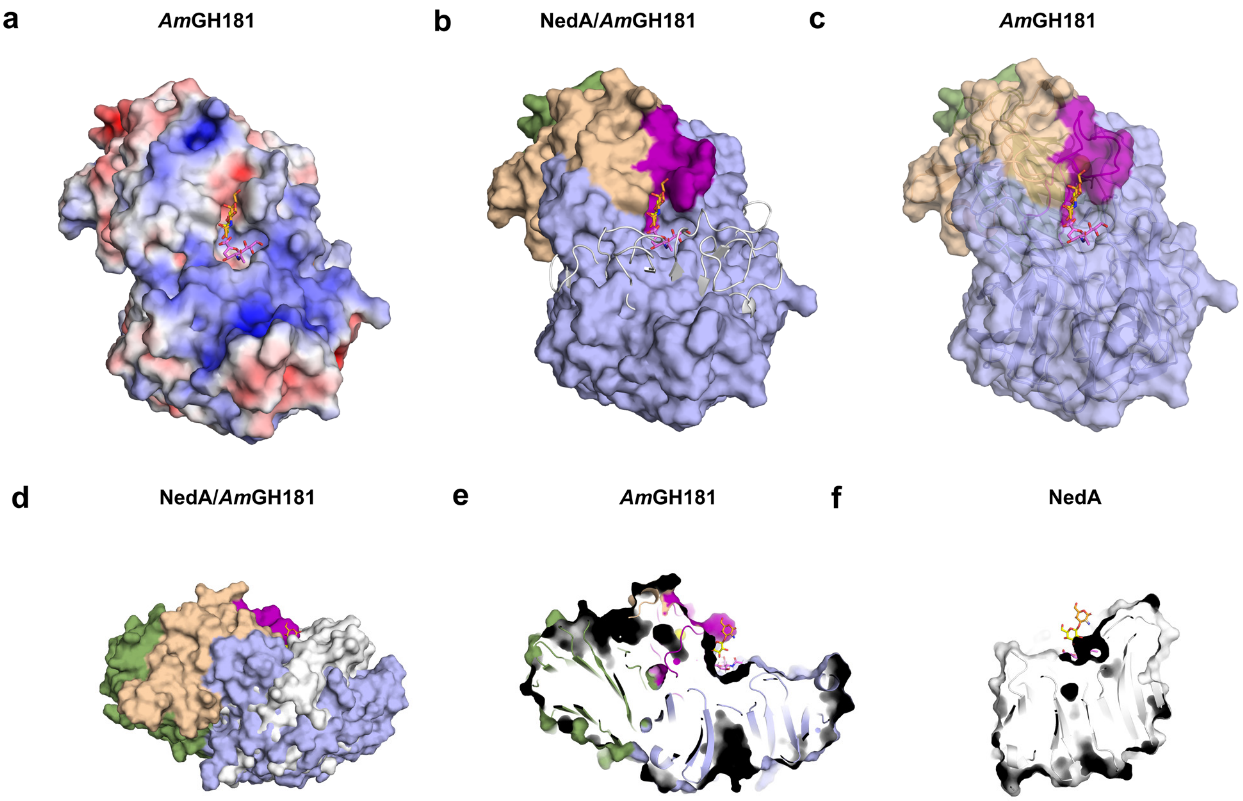

Supplementary Fig. 13: Architecture of *Am*GH181 and comparison to GH33 sialidases. a, Electrostatic surface representation of *Am*GH181 showing positively charged patches surrounding the catalytic site. **b,** Superimposition of *Am*GH181 with the closest structurally characterized orthologue, the GH33 sialidase NedA from *Micromonospora vifidifaciens* (1EUS), showing that the elongated loops that join the strands of the β-propeller in GH33 (grey cartoon) are markedly shortened in *Am*GH181, resulting in open side of the active site as opposed to NedA. By contrast, longer loops together with the Ca^{2+} binding and the B domains pack onto the β-propeller forming the binding site for the T-antigen disaccharide moiety, which lacks in GH33 enzymes. *Am*GH181 is shown as a surface coloured according to the domains: catalytic domain in light blue, Ca^{2+} (yellow sphere) binding domain in violet, B domain in wheat and C-terminal β-sandwich domain in green. **c,** Semi-transparent surface representation of *Am*GH181 coloured as in b showing that the active site of *Am*GH181 is shaped by the Ca^{2+} bindings site, the B domain and two large loops from the catalytic domain. **d,** Superimposition of *Am*GH181 (solid surface, coloured as in **b**) with NedA (solid surface, white) showing differences in active site architecture. **e,** Same view of *Am*GH181 as **d** but represented as carved solid surface (coloured as in **b**) highlighting the "sun-chair" architecture of the *Am*GH181 active site. **f,** Same view of NedA as in **d** but represented as carved solid surface (white) showing the flat surface NedA potentially lacking the aglycone (+) ligand binding sites. Structural alignments of *Am*GH181 and NedA (panels **b** and **d**) were performed using the DALI server.

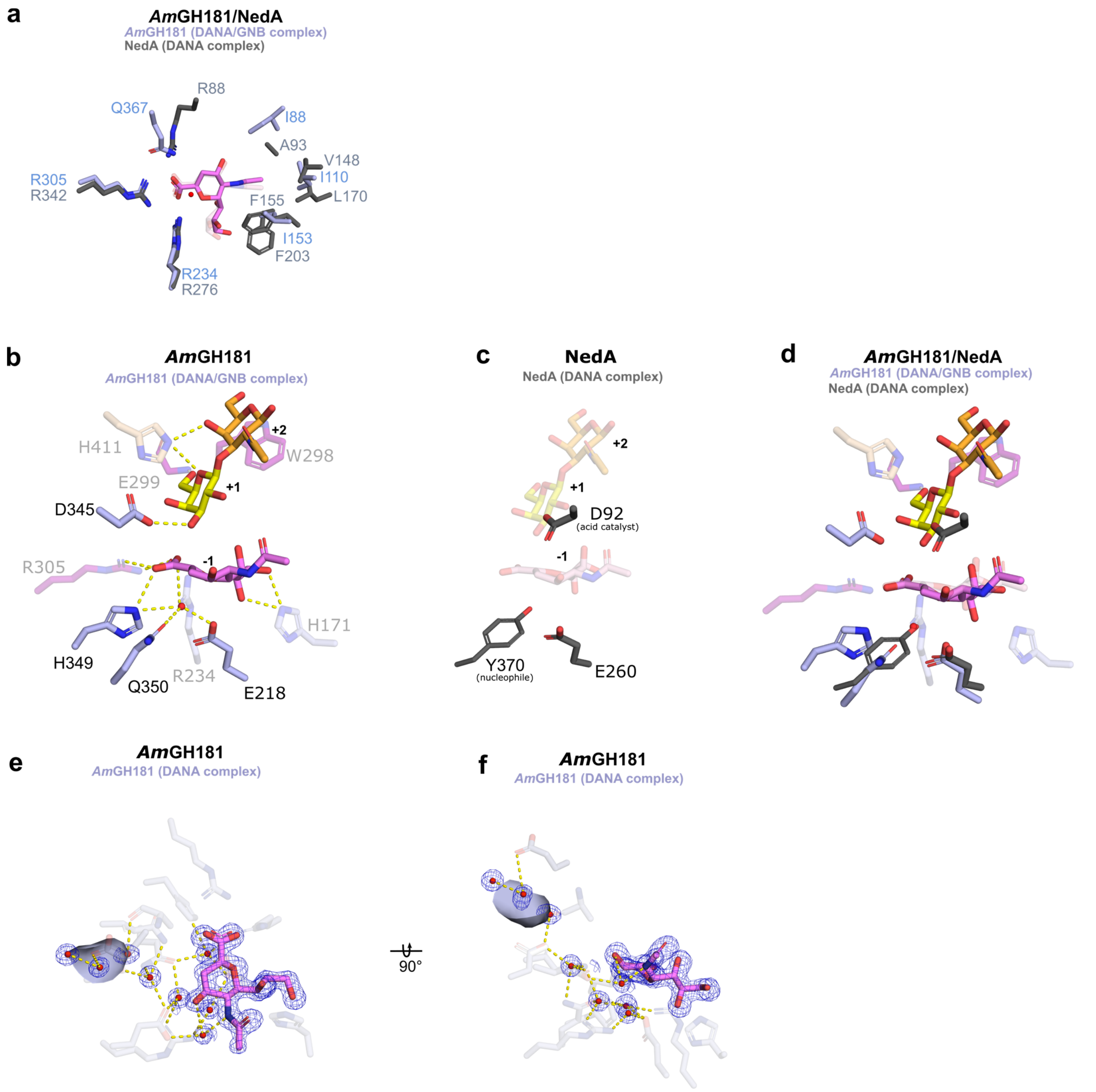

Supplementary Fig. 14 Catalytic site signatures of *Am*GH181 as compared to the closest GH33 sialidase. a, Superimposition of the catalytic sites of *Am*GH181 (light blue sticks, the DANA inhibitor in dark pink, Gal in yellow, GalNAc in dark yellow and an ordered water molecule as red sphere) and NedA, the GH33 from *Micromonospora vifidifaciens* (1EUS, grey sticks, the DANA inhibitor in a semi-transparent and light pink). The top view shows that only two of the three arginine residues of the conserved R triad in GH33, are conserved in *Am*GH181, whereas a glutamine substitutes the third arginine. Residues with similar chemistry flank the *N*-acetyl group of the DANA inhibitor, while two phenylalanines, one of which packs onto the DANA inhibitor in the catalytic site, are lacking in *Am*GH181 **b,** The catalytic site of *Am*GH181 showing the recognition of the DANA inhibitor and T-antigen disaccharide (GNB) (stick coloured according to domain, similar to Supplementary Fig. 11b). **c,** The catalytic machinery of the GH33 NedA (stick representation) showing the catalytic tyrosine nucleophile, an adjacent conserved glutamate and the catalytic acid. The DANA and GNB from *Am*GH181 are also visualized to keep the perspective of the active site. **d,** Superimposition of the *Am*GH181 catalytic site (same colouring scheme as b) and NedA (grey sticks, DANA in semi-transparent and light pink) showing the substitution of the catalytic nucleophile in GH33 to a glutamine (Q350) in *Am*GH181, which is preceded by a histidine (H349). These two residues as well as an invariant glutamate (E218) and one of the conserved arginine (R234) are potentially hydrogen bonded to a water molecule (see b) that overlays perfectly with the oxygen in the catalytic tyrosine in GH33 enzymes. This water is positioned for nucleophilic attack at the C2 of the sialyl (or inhibitor) unit. An invariant aspartate (D345) in *Am*GH181 is hydrogen bonded to the C3-OH group of the bound galactosyl unit at subsite +1 (see b), whereas the general acid/base aspartate in GH33 (D92 in NedA) is missing. **e,** The $F_o - F_c$ electron density maps (blue mesh) of the DANA inhibitor and a solvent tunnel connecting the bulk of the solvent and the catalytic site. **f,** same representation as in e but rotated 90 degrees along the x-axis.

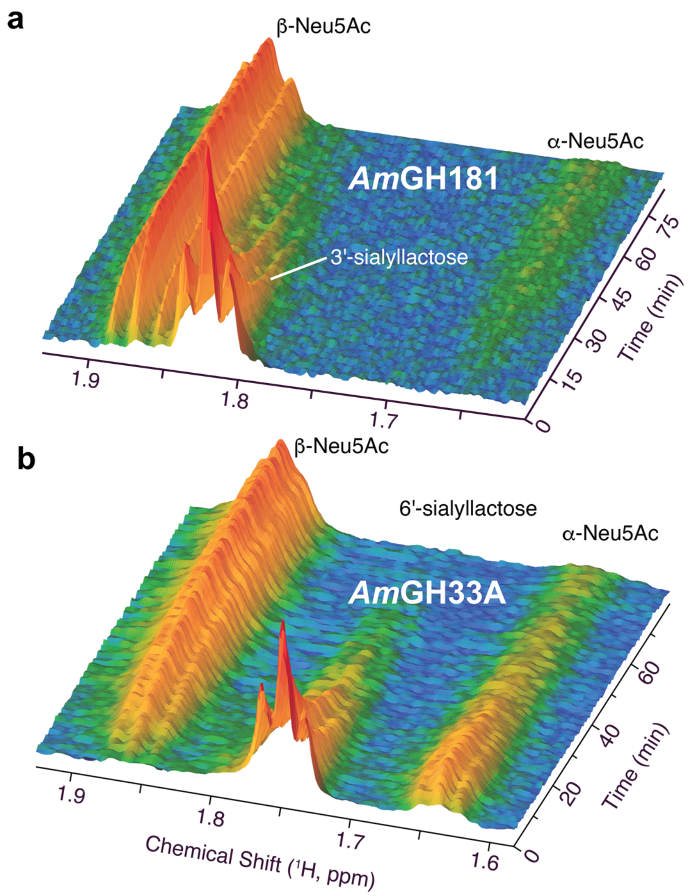

Supplementary Fig. 15: The NMR analysis of the inverting mechanism of *Am*GH181. a, Time series of ^{1}H NMR real time spectra of the *Am*GH181-catalysed conversion of 3´-sialyllactose at 310 K and pH 6.8. The spectral region containing the axial ^{1}H-3 signal in sialic acid (Neu5Ac) is depicted. The spectral series showed that β-Neu5Ac is the initial product of the reaction. Some α-Neu5Ac emerges as the reaction progresses due to mutarotation. These data provide evidence that the hydrolysis of Neu5Ac proceeds with the inversion of anomeric configuration in the previously undescribed GH181 family. **b,** Control experiment that shows a time series of ^{1}H NMR real time spectra of the *Am*GH33B catalysed conversion of 6´-sialyllactose at 310 K and pH 6.8. The spectral region containing the axial ^{1}H-3 signal in sialic acid (Neu5Ac) is depicted. The spectral series showed that α-Neu5Ac is the initial product of the reaction that mutarotates to β-Neu5Ac with a half time of about 80 min to reach about 90% of the total Neu5Ac in the solution. These data are consistent with the known retaining mechanism within GH33. The data are from a single (n=1) experiment. Source data are provided as a Source Data file labelled with the corresponding figure number and panel definition.

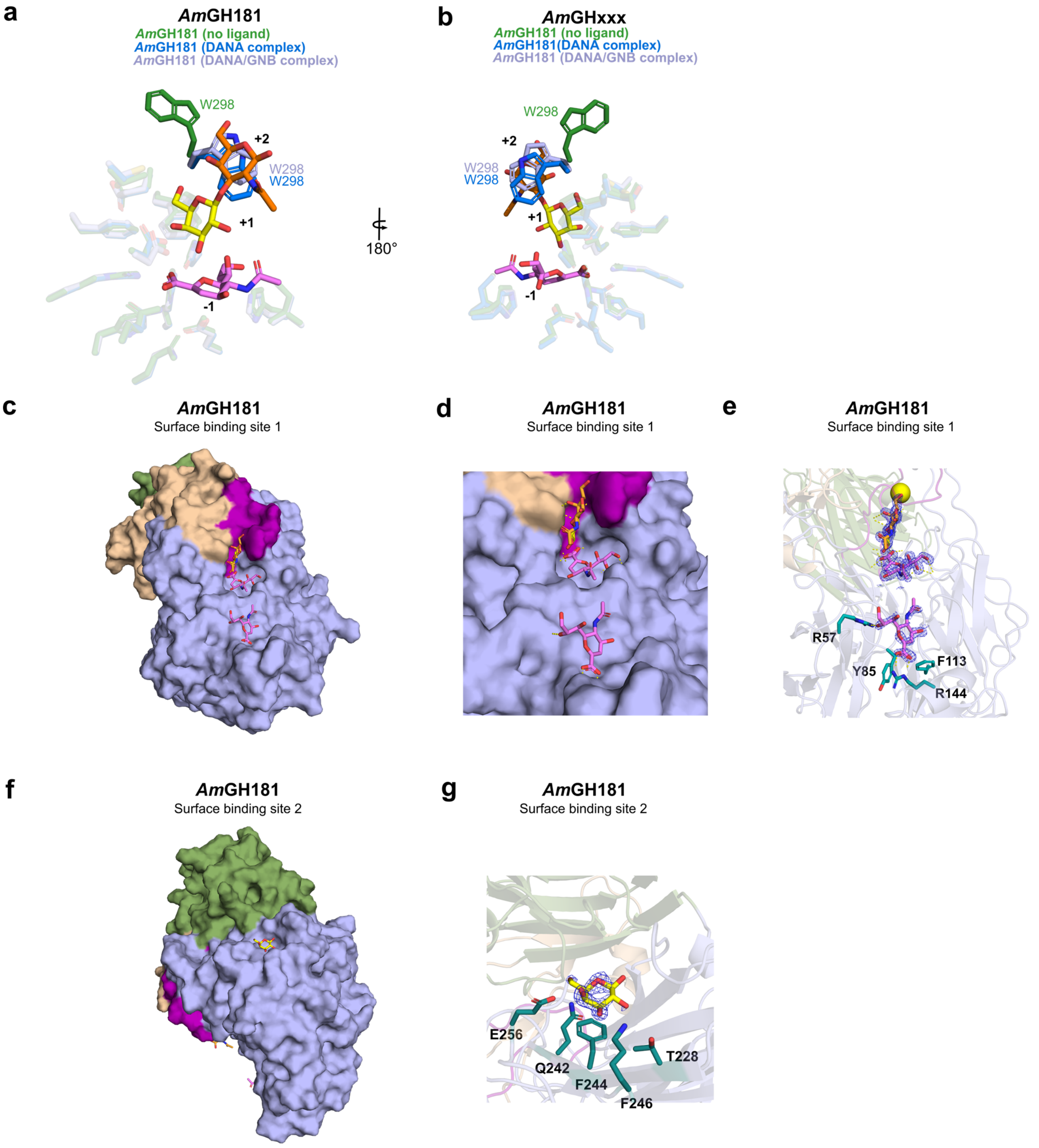

Supplementary Fig. 16: Ligand binding at the active site and secondary surface binding sites of *Am*GH181. a, Comparison of the ligand free (green sticks), DANA bound (blue) or DANA+GNB bound (light purple) structure, showing an induced fit flipping movement of a tryptophan sidechain to provide aromatic stacking for the GalNAc at subsite +2 in the two ligand-bound structures. **b,** The same as **a**, but turned 180°. **c,** Surface binding site 1 adjacent to the active site with a DANA molecule bound. **d,** Zoom in view as in **c**. **e,** potential binding residues at 4 Å distance from the modelled DANA molecule. **f,** The surface binding site 2 at the opposite side of the active site with a modelled galactose unit bound at a shallow groove. **g,** The potential binding resides of the modelled Gal with a phenylalanine aromatic stacking interaction flanked by polar residues. (e and g) Unbiased $F_o - F_c$ electron density maps are represented as blue mesh.

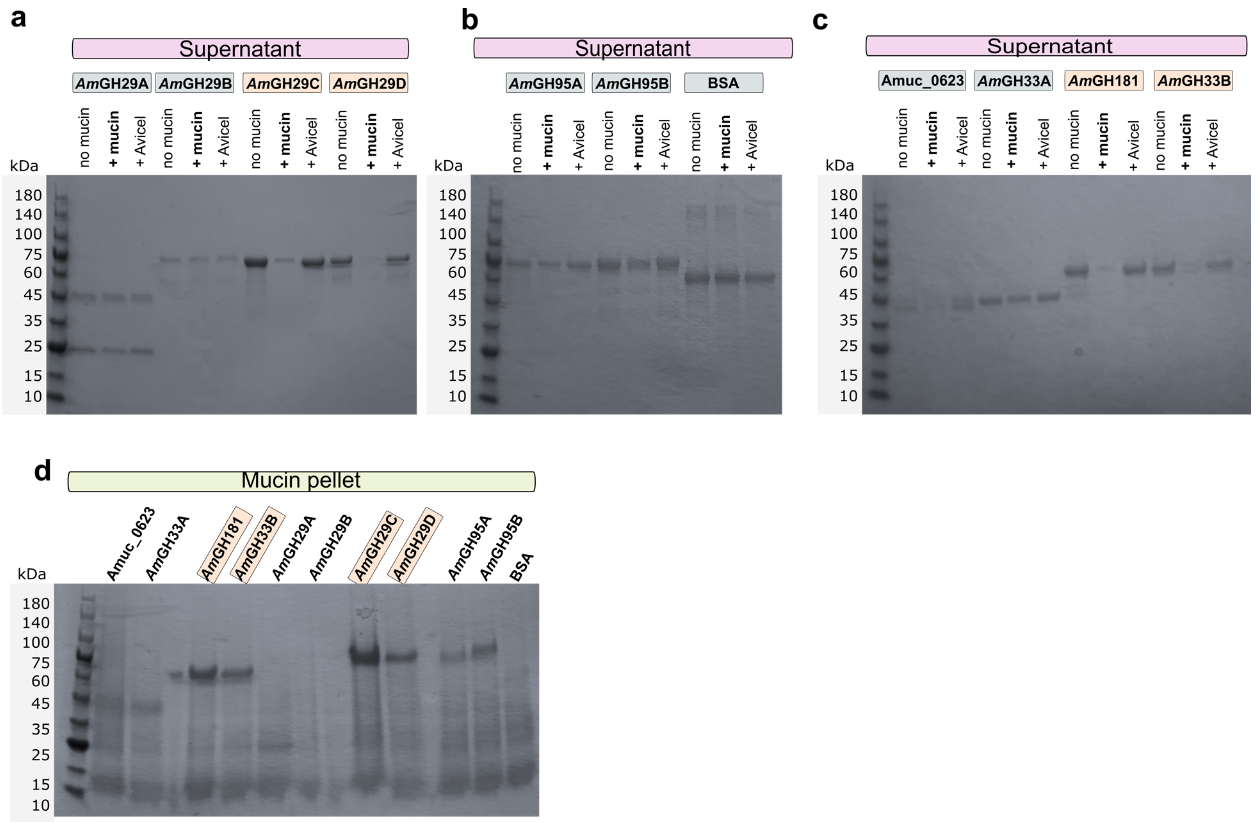

Supplementary Fig. 17: Binding of *A. muciniphila* fucosidases and sialidases to mucin. a-d, Representative SDS-PAGE gels showing pulldown binding assays of *A. muciniphila* fucosidases, sialidases and of a negative control protein (BSA) binding to insoluble PGM and Avicel (about 60% crystalline cellulose) is used as a negative control. A marked decrease in residual enzyme in the supernatant of the binding assay (decreased band intensity) is indicative of the strong association of the enzyme to the insoluble mucin pellet, which is also evidenced by high recovery of the enzyme from the pellets of the binding assays. The data are from two independent experiments (n=2) whereby all analyses yielded similar results. The grey boxes denote enzymes that showed weak or no binding, whereas the bisque coloured boxes denote enzymes that had strong binding to mucin, based on their depletion from the supernatants (pink boxes) of the binding assays. The binding is also investigated by evaluating the proteins in the pellets of the binding assay (green box) that contained the insoluble mucin and the proteins associated to it. The same bisque colour is also used in the pellet fraction to denote strong association, based on the recovery of the bound enzymes from the pellet fraction. The GH95 proteins appear to display some binding, but it is clearly much weaker than counterparts that are highlighted as tight binders in this analysis. Source data are provided as a Source Data file labelled with the corresponding figure number and panel definition.

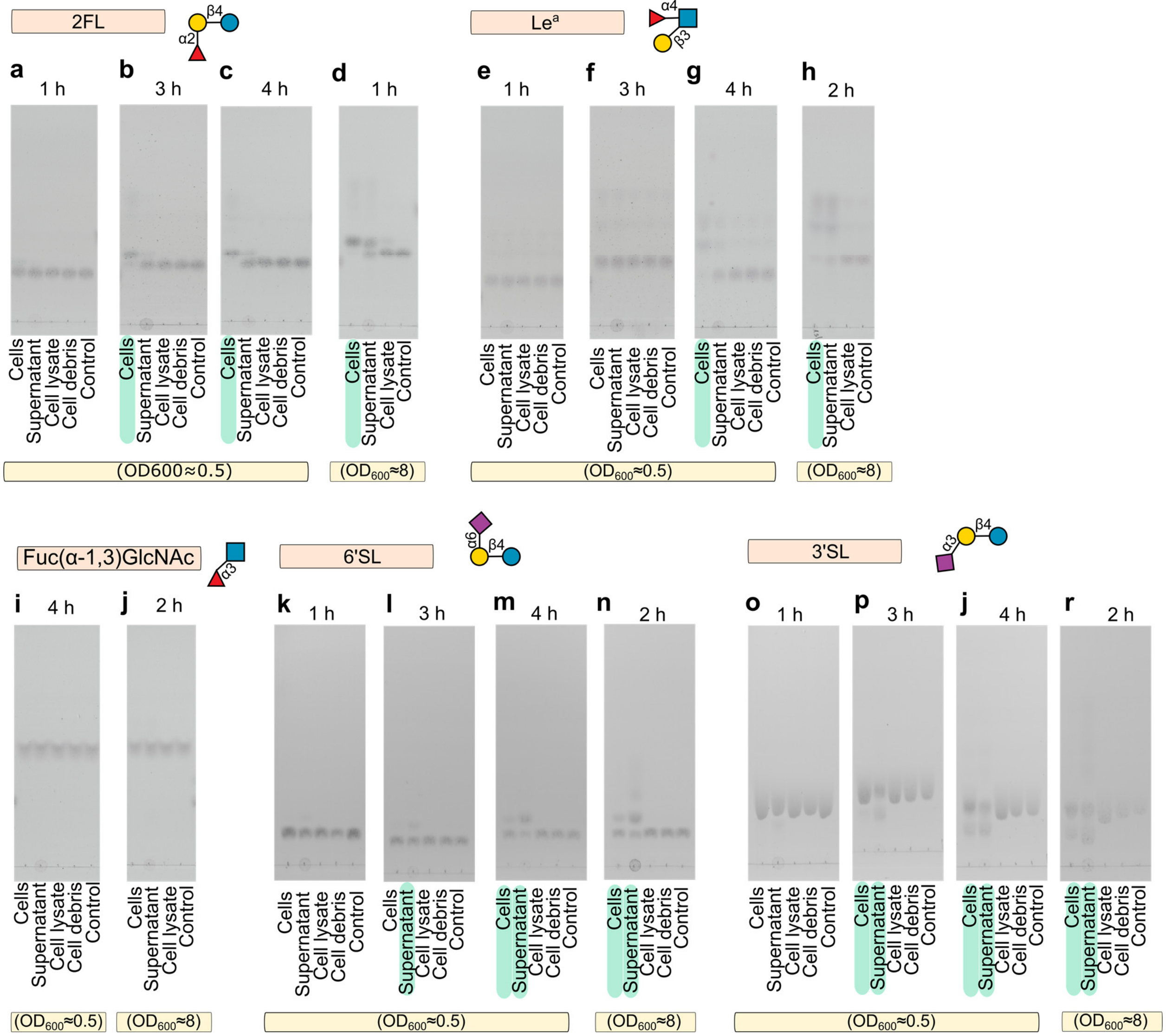

Supplementary Fig. 18: Localization of fucosidase and sialidase activities encoded by *A. muciniphila*. a-d, α1,2-Fucosidase activity of *A. muciniphila* as assayed on 2FL with intact cells, culture supernatants, soluble cell lysates, insoluble cell debris fraction and a buffer control using TLC analysis. **e-h,** α1,4-Fucosidase activity of *A. muciniphila* as assayed on Le^a trisaccharide on similar cell culture fractions as above with a buffer as a negative control. **i-j**. α1,3-Fucosidase activity of *A. muciniphila* as assayed on Fuc α1,3 GlcNAc using same culture fractions and control as above. **k-n,** α2,6-Sialidase activity of *A. muciniphila* as assayed on 6´SL with intact cells, culture supernatants, cell lysates, cell debris and a buffer control. **o-r,** α2,3-Sialidase activity of *A. muciniphila* as assayed on 3´SL with intact cells, culture supernatants, cell lysates, cell debris and a buffer control. The experiments have been performed on cells grown porcine gastric mucin and pure oligosaccharide substrates. Fractions showing highest activity are highlighted with green boxes. The data are from at least three independent experiments (n=3) whereby all analyses yielded similar results. The pellets were resuspended in the same volume as that of the reactions with the supernatants to allow direct comparison. Source data are provided as a Source Data file labelled with the corresponding figure number and panel definition.

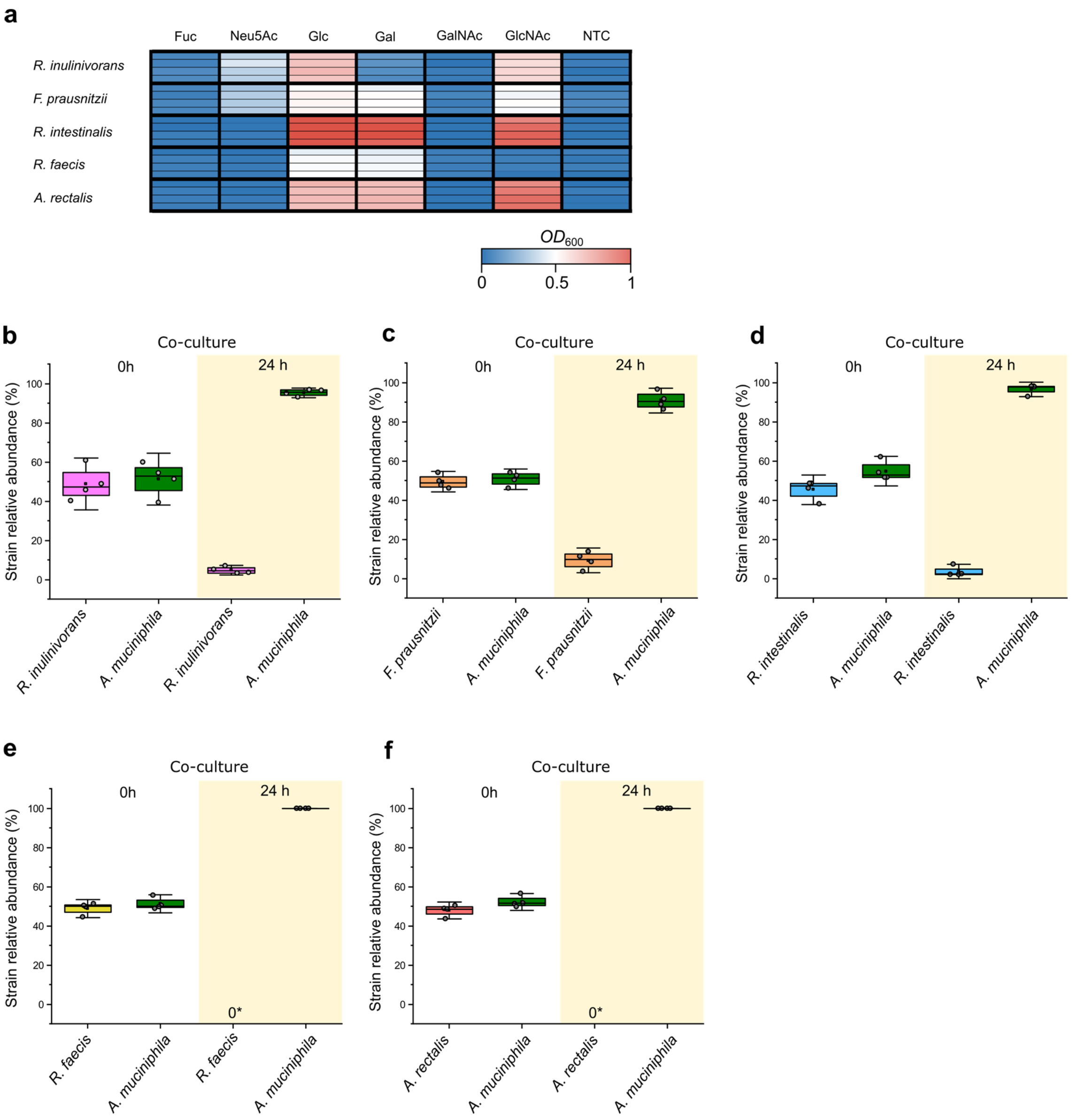

Supplementary Figure 19: Growth of butyrate producing Clostridia on monosaccharides from mucin and in competition with *A. muciniphila* on PCM. a, Growth of *Roseburia inulinivorans, Faecalibacterium prausnitzii*, *Roseburia intestinalis*, *Roseburia faecis* and *Agathobacter rectalis,* on YCFA supplemented with 0.5 % (w/v) monosaccharides from mucin after 24h. Growth experiments were performed in four independent biological replicates (n=4). **b-f,** The relative abundance of *R. inulinivorans*, *F. praunsnitzii* , *R. intestinalis*, *R. faecis*, and *A. rectalis*, respectively, at the start of co-culture growth with *A. muciniphila* on PCM (0 h) and at 24 h. Relative strain abundances were determined with qPCR (one analysis per biological replicate, n=1) from the four independent biological replicate co-cultures presented in Fig. 4 (n=4). In the boxplot, the box represents the interquartile range (25th-75th percentile) and the whisker is 1.5 times the standard deviation (SD). The median is represented as solid line and the mean as a solid black square, with individual measurements are grey circles. Source data are provided as a Source Data file labelled with the corresponding figure number and panel definition.

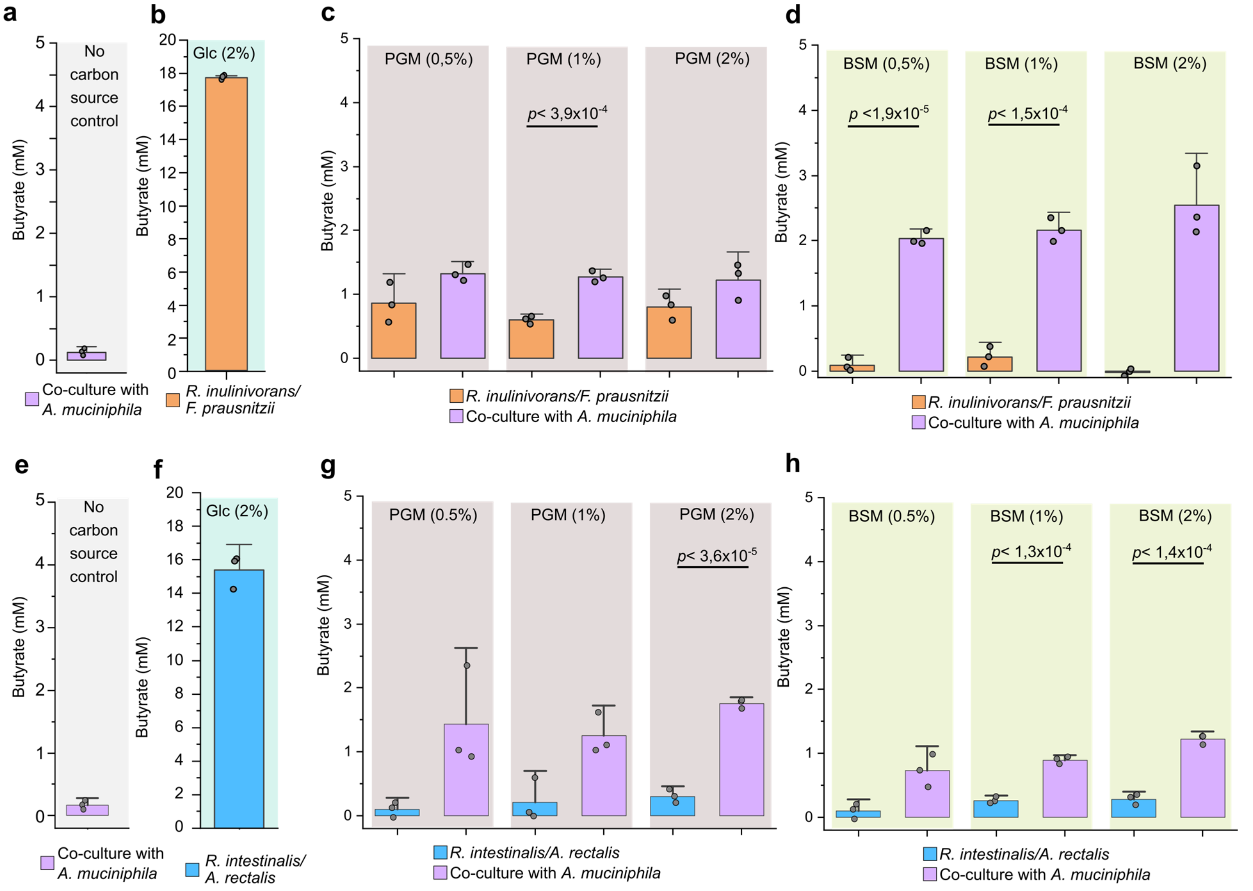

Supplementary Figure 20: Cross-feeding of butyrate producing Clostridia in *A. muciniphila* co-cultures on mucins with different degree of sialyation. a, Butyrate concentration of co-cultures of *R. inulinivorans*, *F. prausnitzii* and *A. muciniphila* grown on a no-carbon source control after 24h. **b,** same as a, but with glucose (2%) as a carbon source. **c,** Butyrate concentration of *R. inulinivorans* and *F. prausnitzii* only (orange) or in co-culture with *A. muciniphila* (purple) on a different concentrations of PGM after 24 h. **d.** Butyrate concentration of *R. inulinivorans* and *F. prausnitzii* only (orange) or in co-culture with *A. muciniphila* (purple) on a different concentration of BSM after 24 h. **e,** Butyrate concentration of co-cultures of *R. intestinalis*, *A. rectalis* and *A. muciniphila* on a non carbon source control after 24h. **f,** Butyrate concentration of *R. intestinalis* and *A. rectalis* only on glucose (2%) after 24 h. **g,** Butyrate concentration of *R. intestinalis* and *A. rectalis* only (blue) or in co-culture with *A. muciniphila* (purple) on a different concentrations of PGM after 24 h. **h.** Butyrate concentration of *R. intestinalis* and *A. rectalis* only (blue) or in co-culture with *A. muciniphila* (purple) on a different concentration of BSM after 24 h. **a-h,** Growth cultures and butyrate quantifications were performed in independent biological triplicates (n=3) and data are presented as mean values with the error bars representing the standard deviation (SD). The statistical significance between butyrate concentrations reached was evaluated using an unpaired two-tailed Student's *t*-test and the corresponding *p*-values are included in the individual figure panels. Source data are provided as a Source Data file labelled with the corresponding figure number and panel definition.

Supplementary Table 1: Enzyme names and primers

Locus tag	SP[a]	Enzyme	GenBank	Sense primer	Antisense primer
			Cloning primers		
Amuc_0010	20	*Am*GH29A	ACD03857.1	**AGGAGATATACCATG**CAGTCCGC CACTAAAATCATTACG	**GGTGGTGGTGCTCGA**GTTTGCT CAGTTTGATGACGGAAC
Amuc_0146	23	*Am*GH29B	ACD03990.1	**AGGAGATATACCATG**GGGAATGC CATCACCGTCC	**GGTGGTGGTGCTCGA**GTTGAAG TTTGATGACGGTATCCAGC
Amuc_0392	36	*Am*GH29C	ACD04231.1	**AGGAGATATACCATG**GGCTGGAC CGCAGCACC	**GGTGGTGGTGCTCGA**GTTTGCC TGCGGGAGTGC
Amuc_0846	24	*Am*GH29D	ACD04679.1	**AGGAGATATACCATG**GGGCCGA AGGGCTGTTTAAC	**GGTGGTGGTGCTCGA**GTTTTCC CAATACGCCCAGCTC
Amuc_0186	23	*Am*GH95A	ACD04030.1	**AGGAGATATACCATG**GCCATTCC GGCCCCCATG	**GGTGGTGGTGCTCGA**GATGGG AAAGCGGAGGAAAATCAAG
Amuc_1120	13	*Am*GH95B	ACD04946.1	**AGGAGATATACCATG**AGTGCCGT TTCTTTCGGGTGG	**GGTGGTGGTGCTCGA**GCCTGGC CGCGGGCTG
Amuc_0623	21		ACD04460.1	**AGGAGATATACCATG**ACCGTACC GGCCCATTCC	**GGTGGTGGTGCTCGA**GGGGAC GTTTCAGAAGGCGATTAAC
Amuc_0625	38	*Am*GH33A	ACD04462.1	**AGGAGATATACCATG**CAGGAAGA GAAAACCGGTTTCC	**GGTGGTGGTGCTCGA**GCTTGAG AACAGGAGCTTTTTTGC
Amuc_1835	17	*Am*GH33B	ACD05653.1	**AGGAGATATACCATG**GGCAAGG AAAGCTTTGAGCAGG	**GGTGGTGGTGCTCGA**GGCGCG CATTTTTTGCGTTAAG
Amuc_1547	22	*Am*GH181	ACD05368.1	**AGGAGATATACCATG**GCACCCGT TCCGGAAC	**GGTGGTGGTGCTCGA**GCTTCAC CCGGGCATTCAC
			qPCR primers		
**Target bacteria**				**Sense primer**	**Antisense primer**
Akkermansia spp.				GCTCACCAAGGCGATGACGG	TGCTCCCACATGACAGGGGTTT AC
Faecalibacterium spp.				GATGGCCTCGCG TCCGATTAG	CCGAAGACCTTC TTCCTCC
Roseburia spp. and *A. rectalis*				GCGGTRCGGCAAGTCTGA	CCTCCGACACTCTAGTMCGAC

[a]The size of the signal peptide in amino acids as predicted from SignalP (V.5.0) (see Materials and methods section). The cloning vector homologous recombination patches to the cloning cassette of the used pET28a(+) vector are in bold. The *A. muciniphila* sialidases with the locus tags Amuc_0623, Amuc_0625, Amuc_1547 and Amuc_1835 have been referred to as Am0705, Am0707, Am1757 and Am2085 respectively1, in previous work that used fluorescently labelled model substrates to indirectly show sialidase activity.

1. Huang K*, et al.* Biochemical characterization of the neuraminidase pool of the human gut symbiont *Akkermansia muciniphila*. *Carbohydr Res* **415**, 60-65 (2015).

Supplementary Table 2: Kinetics parameters of *A. muciniphila* fucosidases.

	Enzyme					
	***Am*GH29A**	***Am*GH29B**	***Am*GH29C**	***Am*GH29D**	***Am*GH95A**	***Am*GH95B**
k_{cat} (s^{-1})	43 ± 1.5	0.10 ± 0.0062	(9.9 ± 0.39) x10^{-3}	0.022 ± 0.0010	0.98 ± 0.017	0.37 ± 0.017
K_M (mM)	1.1 ± 0.11	1.6 ± 0.33	4.1 ± 0.41	2.25 ± 0.34	0.76 ± 0.044	3.0 ± 0.46
k_{cat} /K_M (s^{-1}·mM^{-1})	41 ± 4.6	0.0063 ± 0.0013	0.15 ± 0.0056	0.0096 ± 0.0015	1.29 ± 0.077	0.12 ± 0.020

The kinetic parameters were determined towards the model substrate *para*-nitrophenyl-α-L-Fucoside (*p*NPFuc) at 37 °C for 180 min in 20 mM HEPES, 150 mM NaCl, pH 6.8. Data are mean values of three independent experiments (n=3) with standard deviation (SD). Source data are provided as a Source Data file labelled with the corresponding table number.

Supplementary Table 3: Number of assigned *O*- and *N*-glycan structures studied in this work.

Substrate	Number of assigned glycan structures in the fucosidase analysis: Total	Fucosylated	Sialylated
O-glycans from PGM, PCM and fetuin	160	88	44
N-glycans from human IgG	22	15	10
**Substrate**	**Number of assigned glycan structures in the sialidase analysis: Total**	**Fucosylated**	**Sialylated**
O-glycans from PCM	80	41	30
O-glycans from $MUC2_{Mouse}$	74	17	36
N-glycans from human IgG	24*	13*	10*

Number of glycan structures that are assigned from the LC-MS analysis of reactions of *A. muciniphila* fucosidases and sialidases on different substrates. *The *N*-glycans are from a different experiment than those analysed for fucosidase activity.

Supplementary Table 4: Fucosidase relative activity towards porcine gastric, colonic mucin and fetuin.

	O-glycans			
**Enzyme**	**non-fucosylated** (%)	**Fuc-α1,2** (%)	**Fuc-α1,2 & α1,3/4** (%)	**Fuc-α1,3/4** (%)
Control	56.6	21.2	10.9	11.3
*Am*GH29A	51.6*	23.4*	12.8*	12.2*
*Am*GH29B	57.4*	21.3	10.6	10.7
*Am*GH29C	69.2	30.2	0.3	0.3
*Am*GH29D	59.9	29.3	4.8	6.0
*Am*GH95A	58.7	17.9	10.9	12.6
*Am*GH95B	74.3	2.5	0	23.2

Abundance (%) of non-fucosylated and fucosylated *O*-glycans present in a 1:1:1 per weight mixture of porcine gastric mucin, porcine colonic mucin and fetuin before and after incubation with *A. muciniphila* fucosidases or with buffer as control. Abundances are calculated based on relative intensities of 166 assigned individual glycan structures detected by LC-ESI/MS. Data are from a single (n=1) experiment with enzymatic overnight incubations. The relative abundances were calculated by integration of the LC-ESI/MS ion chromatogram peak (area under the curve, AUC) and the data in the column are the summed relative abundances for each *O*-glycan category. *The changes observed for these enzymatic incubations as compared to the control, reflect the noise of the experiment due to minor differences in the amounts of mucin blots used to assay activity.

Supplementary Table 5: Normalized activity of *A. muciniphila* fucosidases on HMOs, mucins and fetuin.

Substrate	Normalised activity (s^{-1})					
	***Am*GH29A**	***Am*GH29B**	***Am*GH29C**	***Am*GH29D**	***Am*GH95A**	***Am*GH95B**
HMOs	0.63 ± 0.092	0.084 ± 0.011	4.5 ± 0.43	4.0 ± 0.026	3.9 ± 0.078	4.0 ± 0.13
Fetuin	N.D.	N.D.	N.D.	N.D.	N.D.	N.D.
PGM	0.028 ± 0.002	0.019 ± 0.0015	$(59 \pm 0.41)\text{x}10^{-3}$	0.045 ± 0.0014	0.36 ± 0.0041	4.4 ± 0.085
PCM	$(5.8 \pm 0.074)\text{x}10^{-3}$	$(6.6 \pm 0.23)\text{x}10^{-3}$	1.1 ± 0.029	0.46 ± 0.018	0.095 ± 0.014	0.79 ± 0.047

Normalized activity (*V*/E) determined towards 0.5 % (w/v) substrate concentration and a with an enzyme concentration of 0.5 µM. Enzymatic reactions were performed for 1 h, except for reactions containing fetuin which were incubated for 3 h. N.D. not detected. The very low activity of *Am*GH29A and *Am*GH29B on HMOs is likely attributed to activity on the trisaccharide 2'-fucosyl lactose (2FL, see Supplementary Fig. 4e). Data are mean values of three independent experiments (n=3) with standard deviation (SD). Source data are provided as a Source Data file labelled with the corresponding table number.

Supplementary Table 6: Activity profiles of *A. muciniphila* sialidases on porcine colonic mucin *O*-glycans.

	***O*-glycans**			
**Enzyme**	**non-sialylated** (%)	**α2,3** (%)	**α2,3 & α2,6** (%)	**α2,6** (%)
Control	72.2	7.0	1.8	19
Amuc_0623	75.3*	6.0*	2.1*	16.6*
*Am*GH33A	96.9	3.1	0	0
*Am*GH33B	99.3	0.7	0	0
*Am*GH181	75.6	2.6	0.4	21.4

Relative abundance (%) of non-sialylated and differentially sialylated *O*-glycans from porcine colonic mucin before and after incubation with *A. muciniphila* sialidases and or with buffer as control. The relative abundances were calculated based on relative intensities of 82 assigned individual glycan structures detected by LC-MS. The data are for both Neu5Ac and Neu5Gc forms of sialic acid that are cleaved by the enzymes. Data are from a single (n=1) experiment and an overnight incubation. *The differences observed for these enzymatic incubations as compared to the control, reflect the noise due to minor differences in the amounts of mucin blots used to assay activity.

Supplementary Table 7: Normalised activities of *A. muciniphila* sialidases on HMOs and attached *O*-glycans from mucin and fetuin.

Substrate	Sialidase normalised activity (s^{-1})			
	**Amuc_0623**	***Am*GH33A**	***Am*GH181**	***Am*GH33B**
HMOs	$(3.4 \pm 0.46)x10^{-3}$	0.17 ± 0.015	$(13 \pm 3.1)x10^{-3}$	0.18 ± 0.022
Fetuin	$(35 \pm 6.1)x10^{-3}$	0.15 ± 0.02	$(75\pm 36)x10^{-3}$	0.21 ± 0.050
PGM	N.D.	0.014 ± 0.0045	N.D.	$(36 \pm 4.1)x10^{-3}$
PCM	$(5.88 \pm 2.1)x10^{-3}$	0.019 ± 0.0019	$(13 \pm 3.0)x10^{-3}$	$(37 \pm 6.1)x10^{-3}$

Normalized activity (*V*/E) were determined towards 0.5 % (w/v) substrate concentration and with an enzyme concentration of 0.5 µM as measured by the release of sialic acid using HPAEC-PAD. N.D. not detected within 1 h or 3 h assays. Activity of *Am*GH181 on HMOs likely attributed to the very low activity on 3'-Sialyl lactose. Data are means of triplicates (n=3) with standard deviation (SD). Source data are provided as a Source Data file labelled with the corresponding table number.

Supplementary Table 8: The top structural orthologues of *Am*GH29D.

	PDB ID	Z-score	RMSD (Å)	Aligned[a]	Total[b]	Identity (%)	name/GH/Source organism
1	6OR4-B	50.9	1.5	438	449	39	SpGH29/GH29/*Streptococcus pneumoniae* TIGR4
2	5K9H-A	50.3	7.5	457	554	39	GH29_0940/GH29/Rumen unknown bacteria
3	3UES-A	48.8	1.5	433	457	41	BiAfcB/GH29/*Bifidobacterium longum* subsp. infantis ATCC 15697
4	4OZO-B	48.1	1.8	439	459	40	BT2192/GH29/*Bacteroides thetaiotaomicron* VPI-5482
5	4zrx-A	45.5	3.0	451	581	42	Bovatus_01698/GH29/ *Bacteroides ovatus* ATCC 8483
6	6tr3-A	42.6	2.2	448	505	34	CDL26_02305/GH29/*Ruminococcus gnavus* GH29 fucosidase E1
7	3gza-b	41.5	1.9	396	431	30	BT3798/GH29/*Bacteroides thetaiotaomicron* VPI-5482
8	6gn6-C	33.5	2.8	301	421	22	aLfuk1/GH29/*Paenibacillus thiaminolyticus*
9	6o1j-A	32.6	2.0	272	329	25	BN194_28780/GH29/*Lacticaseibacillus casei* W56
10	4jfs-B	31.4	2.7	307	437	26	BtFuc2970/GH29/

The data are based on a DALI search and only the top hit of mutants and/or complexes with ligands is included to avoid redundancy. [a]Aligned residues between the hit protein and *Am*GH29D. [b]Total number of residues in the hit protein.

Supplementary Table 9: The top structural orthologues of *Am*GH181.

	PDB ID	Z-score	RMSD (Å)	Aligned[a]	Total[b]	Identity (%)	name/GH/Source organism
1	1W8O-A	26.2	3.6	311	601	16	NedA sialidase/GH33 *Micromonospora viridifaciens*
2	1SNT-A	26.1	2.8	281	352	10	Neu2 sialidase/GH33 *Homo sapien*
3	2VK7-B	25.6	2.9	283	448	14	NanI sialidase/GH33 *Clostridium perfringens*
4	3H73-B	25.4	3.0	286	477	16	NanA sialidase/GH33 *Streptococcus pneumoniae*
5	5HX0-B	24.4	3.0	279	364	24	Unknown protein/GH33 Dyadobacter fermentans
6	4XJZ-A	23.9	3.1	280	658	20	NanB sialidase/GH33/ *Streptococcus pneumoniae*
7	2SLI-A	23.8	3.0	267	679	18	Intramolecular transsialidase L (MDSA)/GH33/*Macrobdella decora*
8	4X47-A	23.5	3.0	275	489	20	Anhydrosialidase RgNanH/GH33/*Ruminococcus gnavus*
9	6MYV-A	23.3	3.0	277	522	20	Sialidase/GH33/unidentified bacterium
10	4YZ2-A	23.2	2.9	272	655	19	NanC sialidase/GH33 *Streptococcus pneumoniae*

Based on a DALI search; only the top hit of mutants and/or complexes with ligands is included to avoid redundancy. [a]Aligned residues between the hit protein and *Am*GH33B. [b]Total number of residues in the hit protein.

Supplementary Table 10: The top structural orthologues of the CBM-like domain of *Am*GH181.

	PDB ID	Z-score	RMSD (Å)	Aligned[a]	Total[b]	Identity (%)	name/GH/Source organism
1	5MQR-A	15.4	2.1	127	1082	15	BT1020/GH33/*Bacteroides thetaiotaomicron ATCC 29148*
2	4AGG-A	12.9	2.8	121	144	11	Galectin/unknown *Cinachyrelle* sp./marine sponge
3	4HLO-A	12.6	2.2	114	278	7	Galectin/*Tocascaris leonine*/helminth parasite
4	3AFK-A	12.5	2.8	128	168	11	Galectin/*Cyclocybe aegerita*/fungi
5	5XRM-A	12.3	2.6	117	141	18	Galectin/Homo sapiens
6	2A6Y-A	11.5	3.1	140	231	9	Carbohydrate recognition domain/*Saccharomyces cerevisiae*
7	2WSU-B	11	2.5	117	306	10	Galectin/ porine adenovirus 4
8	5N8K-A	10.7	2.6	123	644	11	β-galactocerebrosidase/*Mus musculus*
9	1KIT-A	10.7	2.6	119	757	11	GH33/*Vibrio cholerae*
10	3WUC-B	10.7	2.6	113	137	14	Galectin/*Xenopus laevis*

The data are based on a DALI search and only the top hit of mutants and/or complexes with ligands is included to avoid redundancy. [a]Aligned residues between the hit protein and CBM-like domain of *Am*GH181. [b]Total number of residues in the hit protein.

Supplementary Table 11: Inhibition of *A. muciniphila* fucosidases by 1-Deoxyfuconojirimycin (DFJ).

	Enzyme					
	***Am*GH29A**	***Am*GH29B**	***Am*GH29C**	***Am*GH29D**	***Am*GH95A**	***Am*GH95B**
IC_{50} (µM)	0.60 ± 0.02	1.50 ± 0.10	13.7 ± 0.41	7.29 ± 0.16	53.7 ± 5.9	24.0 ± 1.6

The inhibition constant IC_{50} was determined towards 2 mM *p*NPFuc substrate concentration with inhibitor concentrations in the 0.1 - 100 µM range. The enzyme concentration=0.5 µM for all fucosidases except for *Am*GH29C and *Am*GH29D, which were assayed at concentration= 10 µM due to their low activity. Data are the means of three independent experiments (n=3) with standard deviation (SD). Source data are provided as a Source Data file labelled with the corresponding table number.

Supplementary Table 12: Inhibition of *A. muciniphila* sialidases by 2,3-dehydro-2-deoxy-*N*-acetylneuraminic acid (DANA).

	Enzyme			
	**Amuc_0623**	***Am*GH33A**	***Am*GH33B**	***Am*GH181**
IC_{50} (µM)	N.D.	61.4 ± 2.20	133 ± 11.1	199 ± 13.2
Normalized activity (Emission units s^{-1} nM^{-1})	$(3.7 \pm 0.067)x10^{-3}$	$(108 \pm 6.8)x10^{-3}$	$(74 \pm 2.2)x10^{-3}$	$(36 \pm 1.7)x10^{-3}$

IC_{50} constants determined towards 1 mM 4-Methylumbelliferyl *N*-acetyl-a-D-neuraminic (4MU-Neu5Ac) with an inhibitor concentration in the 0.01-1 mM range. The enzyme concentration was 50 nM for all sialidases, except Amuc_0623 which was assayed at 200 nM. Enzymatic reactions were performed for 30 min and the N.D. is not determined due to the low affinity towards DANA reflected by the lack of curvature, which precluded reliable determination of inhibition constant. The normalized activity, expression in arbitrary emission units per min and nM enzyme is also shown, to depict that the enzymes have different activity levels on this substrate. Data are means of three independent experiments (n=3) with standard deviation (SD). Source data are provided as a Source Data file labelled with the corresponding table number.

Supplementary Table 13: The effects of fucosidase and sialidase inhibition on growth of *A. muciniphila* on PCM.

		Growth substrate			
Time (h)		PCM	PCM + Inhibitors	GlcNAc+GalNAc	GlcNAc+GalNAc+ Inhibitors
5[a]	OD_{600}	0.41 ± 0.01	0.14 ± 0.01	0.21 ± 0.01	0.20 ± 0.01
	p-value	$P < 1.1 \times 10^{-8}$		$P < 0.62$	
8[a]	OD_{600}	1.11 ± 0.04	0.19 ± 0.02	0.39 ± 0.02	0.40 ± 0.05
	p-value	$P < 8.59 \times 10^{-9}$		$P < 0.58$	
24[a]	OD_{600}	1.39 ± 0.04	0.46 ± 0.03	1.32 ± 0.02	1.32 ± 0.04
	p-value	$P < 1.42 \times 10^{-8}$		$P < 0.93$	
24[b]	OD_{600}	1.41 ± 0.02	0.03 ± 0.01	1.43 ± 0.06	1.45 ± 0.05
	p-value	$P < 4.45 \times 10^{-11}$		$P < 0.72$	

Growth level of *A. muciniphila* in the absence or presence of an equimolar fucosidase:sialidase inhibitor blend on PCM or an equimolar mixture of GlcNAc:GalNAc after 5, 8 and 24h. Growth media were supplemented with 0.5% (w/v) carbohydrates and DFJ/DANA to a final concentration of 1 mM[a] or 20[b] mM of each inhibitor. The growth experiments were performed in 4 independent biological replicates (n=4) and the data are presented as mean values with standard deviations. Source data are provided as a Source Data file labelled with the corresponding table number. The statistical significance between *OD600* values reached was evaluated using an unpaired two-tailed Student's *t*-test and the corresponding *p*-values are indicated in the individual table cells.

Supplementary Table 14: Prevalence of fucosidase and sialidase genes in *A. muciniphila* genomes

	Enzyme					
Fucosidase	*Am*GH95A	*Am*GH95B	*Am*GH29A	*Am*GH29B	*Am*GH29C	*Am*GH29D
Prevalence (%)	93.8	98.5	99.4	37.3	99.5	89.9
Sialidase	Amuc_0623	*Am*GH33A	*Am*GH33B	*Am*GH181		
Prevalence (%)	24.4	98.3	98.9	98.3		

Global prevalence of fucosidases and sialidases in 177 *A. muciniphila* genomes of human origin (see materials and methods). The prevalence of fucosidases and sialidases genes were analysed by a BLASTP search using the amino acid sequences of *Am*GH95A, *Am*GH95B, *Am*GH29A, *Am*GH29B, *Am*GH29C, *Am*GH29D, Amuc_0623, *Am*GH33A, *Am*GH33B and *Am*GH181 from *Akkermansia muciniphila* ATCC BAA-835 (same as *Akkermansia muciniphila* DSM 22959) as query.

		Growth substrate			
**Time (h)**		**PCM**	**PCM + Inhibitors**	**GlcNAc/GalNAc**	**GlcNAc/GalNAc + Inhibitors**
5	*OD_{600}*	0.41 ± 0.01	0.14 ± 0.01	0.21 ± 0.01	0.20 ± 0.01
	p-value	$p < 1.1 \times 10^{-8}$		$p < 0.62$	
8	*OD_{600}*	1.11 ± 0.04	0.19 ± 0.02	0.39 ± 0.02	0.40 ± 0.05
	p-value	$p < 8.59 \times 10^{-9}$		$p < 0.58$	
24	*OD_{600}*	1.39 ± 0.04	0.46 ± 0.03	1.32 ± 0.02	1.32 ± 0.04
	p-value	$p < 1.42 \times 10^{-8}$		$p < 0.93$	

		Growth substrate			
**Time (h)**		**PCM**	**PCM + Inhibitors**	**GlcNAc/GalNAc**	**GlcNAc/GalNAc + Inhibitors**
5	*OD600*	0.41 ± 0.01	0.14 ± 0.01	0.21 ± 0.01	0.20 ± 0.01
	p-value	p < 1.1 x 10-8		*p* < 0.62	
8	*OD600*	1.11 ± 0.04	0.19 ± 0.02	0.39 ± 0.02	0.40 ± 0.05
	p-value	p < 8.59 x 10-9		*p* < 0.58	
24	*OD600*	1.39 ± 0.04	0.46 ± 0.03	1.32 ± 0.02	1.32 ± 0.04
	p-value	*p* < 1.42 x 10-8		*p* < 0.93	

Supplementary Table 15: Data collection and refinement statistics of *Am*GH29D

	No additives
PDB accession	8AYR
Resolution range(Å)*	34.45 - 2.70 (2.798 - 2.70)
Space group	P 1
Unit cell (Å, °)	76.19 76.45 84.5 88.41 89.08 88.23
Total reflections	184329 (14917)
Unique reflections	47292 (3963)
Multiplicity	3.9 (3.8)
Completeness (%)	90.33 (75.09)
Mean I/sigma(I)	9.93 (1.63)
Wilson B-factor	59.87
R-merge	0.082 (0.67)
R-meas	0.094 (0.78)
R-pim	0.048 (0.40)
CC1/2	0.99 (0.86)
CC*	0.99 (0.96)
Reflections used in refinement	47185 (3953)
Reflections used for R-free	2450 (205)
R-work	0.23 (0.42)
R-free	0.27 (0.47)
CC(work)	0.95 (0.84)
CC(free)	0.92 (0.72)
Number of non-hydrogen atoms	10626
macromolecules	10602
ligands	4
solvent	20
Protein residues	1353
RMS(bonds, Å)	0.005
RMS(angles,°)	0.86
Ramachandran favoured (%)	91.48
Ramachandran allowed (%)	8.01
Ramachandran outliers (%)	0.52
Rotamer outliers (%)	0.82
Clashscore	10.98
Average B-factor ($Å^2$)	90.90
macromolecules	90.94
ligands	96.59
solvent	68.08
Number of TLS groups	6

*Statistics for the highest-resolution shell are shown in parentheses

Supplementary Table 16: Data collection and refinement statistics of *Am*GH181.

	No ligand	DANA	DANA and GNB
PDB accession	8AXT	8AXS	8AXI
Resolution range(Å)*	53.74 - 1.59 (1.647 - 1.59)	46.35 - 1.3 (1.346 - 1.3)	47.23 - 1.25 (1.295 - 1.25)
Space group	P 1 21 1	P 1 21 1	P 1 21 1
Unit cell(Å, °)	72.96 56.79 146.72 90 94.55 90	104.07 51.78 118.44 90 93.07 90	103.92 51.51 118.47 90 93.15 90
Total reflections	1073418 (104775)	1692885 (126087)	2015188 (138573)
Unique reflections	160820 (15926)	308437 (29552)	323490 (25068)
Multiplicity	6.7 (6.6)	5.5 (4.2)	6.2 (5.5)
Completeness (%)	99.77 (99.70)	99.38 (95.93)	93.50 (72.90)
Mean I/sigma(I)	6.66 (1.35)	11.78 (0.87)	15.68 (2.50)
Wilson B-factor	15.12	16.59	14.13
R-merge	0.29 (>1)	0.063 (>1)	0.049 (0.63)
R-meas	0.32 (>1)	0.069 (>1)	0.053 (0.70)
R-pim	0.123(<1)	0.027 (0.8868)	0.021 (0.28)
CC1/2	0.99 (0.33)	0.999 (0.48)	0.999 (0.87)
CC*	0.997 (0.71)	1 (0.81)	1 (0.96)
Reflections used in refinement	160792 (15926)	307572 (29515)	323214 (25037)
Reflections used for R-free	8029 (805)	15493 (1486)	16213 (1226)
R-work	0.168 (0.29)	0.155 (0.42)	0.153 (0.25)
R-free	0.202 (0.33)	0.175 (0.43)	0.169 (0.27)
CC(work)	0.963 (0.75)	0.975 (0.68)	0.970 (0.92)
CC(free)	0.948 (0.62)	0.969 (0.67)	0.965 (0.90)
Number of non-hydrogen atoms	10349	10778	10756
macromolecules	9014	9212	9057
ligands	7	180	240
solvent	1328	1467	1573
Protein residues	1142	1145	1143
RMS(bonds, Å)	0.010	0.012	0.011
RMS(angles, °)	1.05	1.06	1.11
Ramachandran favoured (%)	96.40	96.49	96.75
Ramachandran allowed (%)	3.43	3.34	3.25
Ramachandran outliers (%)	0.18	0.18	0.00
Rotamer outliers (%)	0.31	0.41	0.31
Clashscore	2.40	2.54	3.07
Average B-factor($Å^2$)	19.75	24.20	21.80
macromolecules	18.48	22.67	19.99
ligands	21.65	31.64	32.39
solvent	28.36	33.29	31.36
Number of TLS groups	11	12	11

*Statistics for the highest-resolution shell are shown in parentheses.
