# Supplementary material for: Sialidases and fucosidases of Akkermansia muciniphila are crucial for growth on mucin and nutrient sharing with mucus-associated gut bacteria
Source: Nat Commun. 2023 Apr 1;14:1833. doi: 10.1038/s41467-023-37533-6 (PMC10067855; doi:10.1038/s41467-023-37533-6)
Supplement: Supplementary file 3 — Description of Additional Supplementary Files [file 41467_2023_37533_MOESM3_ESM.pdf]

Description of Additional Supplementary Files

File Name: Supplementary Data 1
Description: LC-ESI/MS data.

File Name: Supplementary Data 2
Description: LC-ESI/MS data without glycans with relative abundance <0.5%. The latter file is cited only in the SI data per relevance and clarity.
