# Supplementary material for: Sialidases and fucosidases of Akkermansia muciniphila are crucial for growth on mucin and nutrient sharing with mucus-associated gut bacteria
Source: Nat Commun. 2023 Apr 1;14:1833. doi: 10.1038/s41467-023-37533-6 (PMC10067855; doi:10.1038/s41467-023-37533-6)
Supplement: Supplementary file 6 — Reporting Summary [file 41467_2023_37533_MOESM6_ESM.pdf]

nature portfolio

Corresponding author(s): Maher Abou Hachem, Jens Preben Morth

Last updated by author(s): Mar 1, 2023

Reporting Summary

Nature Portfolio wishes to improve the reproducibility of the work that we publish. This form provides structure for consistency and transparency in reporting. For further information on Nature Portfolio policies, see our Editorial Policies and the Editorial Policy Checklist.

Statistics

For all statistical analyses, confirm that the following items are present in the figure legend, table legend, main text, or Methods section.

n/a	Confirmed	
☐	☒	The exact sample size (*n*) for each experimental group/condition, given as a discrete number and unit of measurement
☐	☒	A statement on whether measurements were taken from distinct samples or whether the same sample was measured repeatedly
☐	☒	The statistical test(s) used AND whether they are one- or two-sided<br>*Only common tests should be described solely by name; describe more complex techniques in the Methods section.*
☒	☐	A description of all covariates tested
☒	☐	A description of any assumptions or corrections, such as tests of normality and adjustment for multiple comparisons
☐	☒	A full description of the statistical parameters including central tendency (e.g. means) or other basic estimates (e.g. regression coefficient) AND variation (e.g. standard deviation) or associated estimates of uncertainty (e.g. confidence intervals)
☐	☒	For null hypothesis testing, the test statistic (e.g. *F*, *t*, *r*) with confidence intervals, effect sizes, degrees of freedom and *P* value noted<br>*Give P values as exact values whenever suitable.*
☒	☐	For Bayesian analysis, information on the choice of priors and Markov chain Monte Carlo settings
☒	☐	For hierarchical and complex designs, identification of the appropriate level for tests and full reporting of outcomes
☒	☐	Estimates of effect sizes (e.g. Cohen's *d*, Pearson's *r*), indicating how they were calculated

Our web collection on statistics for biologists contains articles on many of the points above.

Software and code

Policy information about availability of computer code

Data collection	The Topspin 3.5 pl6 has been used for the analysis of the NMR data. The MXCuBE v3 was used at BioMAX/MAX IV.
Data analysis	OriginPro 2021 (academic) has been used for graphics and statistical analyses. The Chromeleon 6.80 SR10 software has been used to analyse HPAEC data. The Topspin 3.5 pl6 has been used for the analysis of the NMR data. The following programs have been used for the structural analyses: The PyMol software version 2.2.0 has been used for the structural analyses and molecular rendering. Alphafold modelling was performed using ColabFold v1.5.2. Phasing was performed with the phaser version in included in the phenix package (v.1.19.2-4158); The structures were refined using phenix.refine included in the phenix package version 1.19.2-4158 and manually rebuilt using Coot61 v0.9. The manual model was analysed using MolProbity, using phenix package version 1.19.2-4158. Identification of closest structural characterized orthologues were done using the Dali server. The Protparam tool was used for protein sequence analysis. SignalP 5.0, PSORTb (v.3.0.3), and TMHMM (v.2.0) were used for signal peptide prediction. BlastP was used for protein sequence search and retrieval. The CD-HIT server has been used to reduce redundancy of sequences. MAFFT was used for sequence alignment. Structure-guided protein sequence alignments were performed using PROMALS3D. The phylogenetic analysis was performed using NGphylogeny and rendered using iTOL v6. The visualization of amino acid conservation was made using Seq2Logo v2.0. The PATRIC database v.3.6.12 was used to search metagenome data.

For manuscripts utilizing custom algorithms or software that are central to the research but not yet described in published literature, software must be made available to editors and reviewers. We strongly encourage code deposition in a community repository (e.g. GitHub). See the Nature Portfolio guidelines for submitting code & software for further information.

Data

Policy information about availability of data

All manuscripts must include a data availability statement. This statement should provide the following information, where applicable:

- Accession codes, unique identifiers, or web links for publicly available datasets
- A description of any restrictions on data availability
- For clinical datasets or third party data, please ensure that the statement adheres to our policy

The atomic coordinates of AmGH29A and AmGH181 have been deposited in the Protein Data Bank (https://www.rcsb.org) under the PDB accessions 8AYR as well as 8XI, 8AXS and 8XT, respectively (see also Supplementary Information Tables 15 and 16). The GenPept accession IDs of the enzymes characterised in the study are enlisted in Supplementary Table 1. All the data are available from the corresponding authors upon request. Source data are provided with this paper as Supplementary Data file.

Human research participants

Policy information about studies involving human research participants and Sex and Gender in Research.

Reporting on sex and gender	Irrelevant as no human participants are included
Population characteristics	Irrelevant
Recruitment	Irrelevant
Ethics oversight	Irrelevant

Note that full information on the approval of the study protocol must also be provided in the manuscript.

Field-specific reporting

Please select the one below that is the best fit for your research. If you are not sure, read the appropriate sections before making your selection.

☒ Life sciences ☐ Behavioural & social sciences ☐ Ecological, evolutionary & environmental sciences

For a reference copy of the document with all sections, see nature.com/documents/nr-reporting-summary-flat.pdf

Life sciences study design

All studies must disclose on these points even when the disclosure is negative.

Sample size	Most analyses were performed in independent triplicate or less frequently duplicate experiments, as deemed sufficient to give a reliable error estimate. The NMR analyses were performed in a single experiment, as the output form this is sufficient to determine the mechanism with the control included. The LC-MS analyses were performed in a single replicate due the large number of glycans analyses, which would include sufficient redundancy to give reliable data. Similarly activity on conjugated P-selectin gave sufficient information to confirm the LC-MS data with regard to enzyme regio-selectivity.
Data exclusions	No data were excluded
Replication	Independent triplicate or less frequently duplicate experiments were performed, except for the NMR and the LC-ESI/MS experiments, as explained above. This is indicated in Table and Figure legends.
Randomization	No animal or human subjects were involved, and the randomisation of data was not relevant.
Blinding	No animal or human subjects were involved, and blinding was not relevant.

Reporting for specific materials, systems and methods

We require information from authors about some types of materials, experimental systems and methods used in many studies. Here, indicate whether each material, system or method listed is relevant to your study. If you are not sure if a list item applies to your research, read the appropriate section before selecting a response.

Materials & experimental systems

n/a	Involved in the study	
☐	☒	Antibodies
☒	☐	Eukaryotic cell lines
☒	☐	Palaeontology and archaeology
☐	☒	Animals and other organisms
☒	☐	Clinical data
☒	☐	Dual use research of concern

Methods

n/a	Involved in the study	
☒	☐	ChIP-seq
☒	☐	Flow cytometry
☒	☐	MRI-based neuroimaging

Antibodies

Antibodies used	Primary antibodies: mouse IgG anti-Lewis antigen (Sigma–Aldrich); IgG anti-Blood Group Lewis A (7LE) (Santa Group Biotechnology, cat log: sc-51512); IgM anti-Blood Group Lewis B (T218) (Santa Group Biotechnology, cat log: sc-59470); IgM anti-Lewis X antibody: CD15 (C3D-1) (Santa Cruz Biotechnology, sc-19648); IgM anti-Blood Group Lewis Y (F3) (abcam, cat log: ab 3359).<br>The secondary antibodies: HRP-conjugated poly-clonal goat anti-mouse IgM (Sigma–Aldrich); goat antibodies for Lewis A: Peroxidase goat anti-mouse IgG, F(ab´)2 (Jackson ImmunoResearch, cat log: 115-035-006) and for Lewis B, X and Y: Peroxidase goat anti-mouse IgM antibody (Sigma-Aldrich A-8786).
Validation	All antibodies were validated by manufacturers.

Animals and other research organisms

Policy information about studies involving animals; ARRIVE guidelines recommended for reporting animal research, and Sex and Gender in Research

Laboratory animals	Irrelevant
Wild animals	Irrelevant
Reporting on sex	Irrelevant
Field-collected samples	Irrelevant
Ethics oversight	Irrelevant

Note that full information on the approval of the study protocol must also be provided in the manuscript.
